# A quantitative analysis of Final Palaeolithic/earliest Mesolithic cultural taxonomy and evolution in Europe

**DOI:** 10.1371/journal.pone.0299512

**Published:** 2024-03-11

**Authors:** Felix Riede, David N. Matzig, Miguel Biard, Philippe Crombé, Javier Fernández-Lopéz de Pablo, Federica Fontana, Daniel Groß, Thomas Hess, Mathieu Langlais, Ludovic Mevel, William Mills, Martin Moník, Nicolas Naudinot, Caroline Posch, Tomas Rimkus, Damian Stefański, Hans Vandendriessche, Shumon T. Hussain

**Affiliations:** 1 Department of Archaeology and Heritage Studies, Aarhus University, Højbjerg, Denmark; 2 INRAP, INRAP Centre Île-de-France Institut National de Recherches Archéologiques Préventives 18 rue Chapelle, Technologie et Ethnologie des Mondes Préhistorique, University of Paris-Nanterre, Nanterre, France; 3 Department of Archaeology, Ghent University, Ghent, Belgium; 4 I.U. de Investigación en Arqueología y Patrimonio Histórico, University of Alicante, Alicante, Spain; 5 Dipartimento di Studi Umanistici – Sezione di Scienze Preistoriche e Antropologiche, University of Ferrara, Ferrara, Italy; 6 Museum Lolland-Falster, Nykøbing F, Denmark; 7 CNRS UMR 5199 PACEA, University of Bordeaux, France & SERP University of Barcelona, Barcelona, Spain; 8 CNRS UMR 7 8068 Technologie et Ethnologie des Mondes PréhistoriqueS, University of Paris-Nanterre, Nanterre, France; 9 Zentrum für Skandinavische und Baltische Archäologie, Schloß Gottorf, Schleswig, Germany; 10 Department of Geology, Faculty of Science, Palacky University Olomouc, Olomouc, Czech Republic; 11 CNRS-CEPAM, Université Côte d’Azur, Nice, France; 12 Natural History Museum Vienna, Vienna, Austria; 13 Institute of Baltic Region History and Archaeology, Klaipėda University, Klaipėda, Lithuania; 14 Archaeological Museum Kraków, Kraków, Poland; 15 Institute for Prehistoric Archaeology, University of Cologne, Cologne, Germany; Universita degli Studi di Ferrara, ITALY

## Abstract

Archaeological systematics, together with spatial and chronological information, are commonly used to infer cultural evolutionary dynamics in the past. For the study of the Palaeolithic, and particularly the European Final Palaeolithic and earliest Mesolithic, proposed changes in material culture are often interpreted as reflecting historical processes, migration, or cultural adaptation to climate change and resource availability. Yet, cultural taxonomic practice is known to be variable across research history and academic traditions, and few large-scale replicable analyses across such traditions have been undertaken. Drawing on recent developments in computational archaeology, we here present a data-driven assessment of the existing Final Palaeolithic/earliest Mesolithic cultural taxonomy in Europe. Our dataset consists of a large expert-sourced compendium of key sites, lithic toolkit composition, blade and bladelet production technology, as well as lithic armatures. The dataset comprises 16 regions and 86 individually named archaeological taxa (‘cultures’), covering the period between ca. 15,000 and 11,000 years ago (cal BP). Using these data, we use geometric morphometric and multivariate statistical techniques to explore to what extent the dynamics observed in different lithic data domains (toolkits, technologies, armature shapes) correspond to each other and to the culture-historical relations of taxonomic units implied by traditional naming practice. Our analyses support the widespread conception that some dimensions of material culture became more diverse towards the end of the Pleistocene and the very beginning of the Holocene. At the same time, cultural taxonomic unit coherence and efficacy appear variable, leading us to explore potential biases introduced by regional research traditions, inter-analyst variation, and the role of disjunct macroevolutionary processes. In discussing the implications of these findings for narratives of cultural change and diversification across the Pleistocene-Holocene transition, we emphasize the increasing need for cooperative research and systematic archaeological analyses that reach across research traditions.

## Introduction

Beginning with designations such as Perigordian/Gravettian, Solutrean and Magdalenian during the earliest days of Palaeolithic archaeology in France, named archaeological cultures (NACs) have played a major role in thinking about when and how hunter-gatherer populations transformed and adapted to the often rapidly changing climate regimes of that time [e.g. 1–3]. The rising number of archaeological discoveries during the 19^th^ and 20^th^ centuries [cf. 4] led many earlier scholars to focus on typological definitions of NACs, which in turn were used in interpretations of cultural history, settlement patterns, migration and cultural ecology [5–9]. NACs were intended to provide order to the wealth of archaeological material, and to facilitate an improved understanding of the observed changes in material culture over time [cf. 10,11]. Clearly, issues of classification and taxonomy—from individual artefacts to large-scale phenomena—present a long-standing concern in archaeology in general [see 12], and in Palaeolithic studies in particular [13–15].

The Final Palaeolithic (~15,000 to 11,500 years ago cal BP), situated during the abrupt climatic and environmental transition from the Pleistocene into the Holocene [16] is not exempt from classificatory debate. Monographs focusing on key cultures, technocomplexes or culture-historical episodes form the backbone of current taxonomic designations [9,17–27], supplemented with contributions presenting regional records published in keystone edited volumes [e.g. 28–43] as well as a plethora of journal articles in a wide array of journals and languages. The result of this long research history is a patchwork of local, regional, national, and supra-national taxonomic units, which are variously shared, ignored or co-opted across different scholarly communities.

This register of cultural taxa includes cultural taxonomic denominations such as the Late Magdalenian [44,45], the Epigravettian [46–50], the Shouldered Point Complex including the Hamburgian with its enigmatic Havelte sub-group (see Schmider [51], Weber [52] and Pedersen et al. [53]) and the Creswellian [54–56], the Arch-Backed Point complex [= Federmessergruppen; see 9,34,57,58] with its purported sub-groupings—the Tjongerian of the Low Countries, the Rissen and Wehlen Groups, and others (see below)–, the Azilian [27,59], the Tanged Point Complex [26,35,60,61], and the various expressions of the incipient Mesolithic, comprising entities such as the Maglemosean, the Beuronian and the Sauveterrian [e.g. 62–65]. The Tanged Point Complex is commonly subdivided into further sub-units characterised by large tanged points [= Bromme, Perstunian and others; but see 66] and younger groups with smaller point forms (= Ahrensburgian, Swiderian). The perspectives encapsulated by the monographic treatments of this period naturally represent individual authors’ attempts at synthesis, yet the taxonomic frameworks proposed and the methodological approaches taken often do not articulate seamlessly: Different authors classify the same material into different cultural taxonomic groups and units, and this is often tied to differential foundational priority given to divergent dimensions of the lithic record, especially with regard to technological vs. typological aspects of the observed variability.

The ‘Flat Blade and Bladelet Technocomplex’ (FBT) recently proposed based on technological studies [67–69] is a good illustration of this difficulty. In defining the FBT, emphasis is placed on widely shared blade core architectures with low longitudinal and transversal convexities aimed at obtaining flat blades, a characteristic originally ascribed to the Laborian and later recognized in the Belloisian [70], the Long Blade Industries [71], as well as possibly in Ahrensburgian [72] and some Maglemosean contexts [73]. Naudinot et al. [74] regard the FBT as a near-continental unit, spanning large parts of northern Europe. Others have emphasized large numbers of specifically configured microliths, especially so-called Zonhoven points, as key definitional features of the so-called Epi-Ahrensburgian [75,76], especially in the Low Countries. Based on the scant dating evidence, the latter is considered as a younger, southwestern expression of the traditional Ahrensburgian [75]. It is, however, currently unclear whether these taxonomic assignments conflict or complement each other and on which spatiotemporal scales such taxonomic designations operate best, i.e. can the above-mentioned NACs be seen as regional variations nested within a high-order FBT cultural taxon? By the same token, it remains unclear to what extent these perspectives are filtered by more general ideas on operative past culture-historical processes, for example Valentin’s [27] ‘globalisation’ of regular blade and bladelet technology at the very end of the Pleistocene. Vitally, such alternative designations are not neutral as they touch upon the normative bases upon which cultural taxonomies are created and deployed in empirical analyses and interpretations. They have implications for how the period is portrayed and what questions can reasonably be asked.

In contrast to the available monographic treatises, the contributions contained in edited volumes and journal articles are inherently more limited in scope. Furthermore, there is often little epistemological alignment between them, and such differences may over time have introduced substantive inter-observer variation in how primary material is presented and published. This situation is further aggravated by the multitude of languages and research traditions at play, and the tumultuous and at times divisive political and intellectual history of Europe throughout the formative 19^th^ and 20^th^ centuries [77–79]. The same semantic labels may in this way signal different processes depending on the regional and research-historical context in which they are mobilized [28–32,34–38,41–43,80].

Aiming to produce interregional syntheses and comparisons on a large spatiotemporal scale, recent critiques have therefore underlined that many of the current cultural taxonomic units are based on different definitional criteria and that their epistemological status at times remains poorly articulated [66,81]. Lithic assemblages can be classified in many ways—by form, function, raw material, technological organization, or any other property that analysts find salient. Classification is a vital analytical step, and always guided by a specific research question. However, when conducting comparative analyses or syntheses it is essential that the units to be compared are constructed in the same manner. Yet, taxonomic uncertainties seem to persist across all levels of observation, from large-scale cultural units to more localized technocomplexes. Maier [44], for instance, has noted the difficulty of distinguishing the Magdalenian from the Epigravettian in Central Europe on typological grounds. Working across Central Europe, Ikinger [57] pointed out shortcomings of long-standing sub-divisions within the *Federmessergruppen* (FMG). At a regional scale, Nielsen [23] revised Bandi’s [82] initial description of the Swiss *Fazies Fürsteiner*, while Sauer [83] raised critical arguments against the southern German *Atzenhofer Gruppe* of Schönweiß [84]. The previously proposed *Ostroměř Gruppe* of Bohemia [85–87] was later renamed Tishnovian [88,89], and both have since been interrogated on source-critical grounds [90]. In relation to the various groups characterised by tanged points, Kobusiewicz [91] questioned the division between Ahrensburgian and Swiderian on grounds of technological similarities, while Winkler’s [61] analysis supports this division. Szymczak [92] and Sulgostowska [93] debated the reality or otherwise of the so-called Perstunian culture; Kobusiewicz [94] and Riede [95] have taken critical stances regarding the Brommean that hinge on the morphology of lithic diagnostics. Such critiques reflect evident difficulties of inter-observer validation across the broader research community and may also pertain to the reliance on secondary lithic data as it is available in published sources.

The taxonomic differentiation of lithic industries postdating the end of the Magdalenian in the Iberian Mediterranean region (Ebro Valley, Catalunya, and along the Mediterranean arch) presents another example of the at times confusing state of archaeological classification practice in the European Final Palaeolithic. In this area, researchers have recently referred to assemblages as reflecting separated cultural entities, namely ‘Epimagdalenian’ or ‘Microlaminar Epipalaeolithic’ depending on different research traditions [cf. 96,97]. Other recent work has used labels such as ‘Sauveterrian’ or ‘Sauveterroid Epipalaeolithic’ in reference to lithic assemblages dated to the onset of the Younger Dryas [96,98], well before the oldest recognition of the Sauveterrian/Sauveterriano NAC in Southern France and Northern Italy where they tend to date to the Preboreal and Boreal [65]. The semantic similarity between these labels may be at odds with the features defining these cultural phenomena.

At present, there is no shared consensus what precisely the defining standards of cultural taxonomic assignment are for the Palaeolithic on the whole, and the Final Palaeolithic/earliest Mesolithic specifically. Besides variability intrinsic to the archaeological record, key issues in these debates are data inconsistencies, as well as variable assumptions relating to and definitions of archaeological ‘cultures’, ‘industries’, ‘groups’, ‘facies’, ‘technocomplexes’. The long-standing issue of lumper vs. splitter proclivities also plays into this debate, as there is no consensus in sight whether the overarching goal should be to collapse regional and sub-regional units into higher-order ones or rather to highlight regional specificities.

The epistemically problematic yet persistent nature of Palaeolithic cultural taxonomy has been pointed out on several occasions [13,99–102] and arguably relates to the mostly implicit status of the generative principles that lead to a given taxonomic structure: how are key declarative terms such as ‘culture’ to be understood in relation to other taxonomic terms and in relation to underlying behavioural and demographic processes? Intended or not, much Palaeolithic cultural taxonomy is burdened with terminological baggage and latent semantic connotations. Working across classificatory systems with units proposed by different workers is further compounded by different priorities with regard to which kinds of information is used to define a given unit (i.e. the presence of presumed artefact diagnostics or, of key technological traits, or the shapes of specific artefact classes) and on which scale of observation (object, site, region) such information is recorded and available. In this context, Reynolds and Riede [15] go as far as to declare Palaeolithic studies to be in a state of crisis based on this proliferation of different theoretical paradigms, methods, analytical scales, and prioritisations. In principle, each site is unique. By the same token, the degree to which sites differ will vary along a multitude of parameters. Grouping these is a vital step in the organisation and presentation of these, by now, very large amounts of archaeological data. We here attempt to address this problem afresh by re-examining the cultural taxonomic structure of the Final Palaeolithic and earliest Mesolithic on a large geographic, pan-European scale, drawing on a collaboratively assembled multi-dimensional lithic dataset that allows us to quantitatively assess a range of shared NAC attributions and denominations for their compositional integrity, definitional consistency, and discriminatory power.

Attempts to construct cultural taxonomic schemes for the Palaeolithic/Mesolithic using computational approaches such as cluster, factor, or network analysis have been undertaken previously [57,103–108], and we here build on these efforts. Our analysis aims to evaluate the efficacy and replicability of contemporary cultural classifications of the Final Palaeolithic and the earliest Mesolithic in Europe. In seeking to benchmark classifications across research traditions, regions and analysts, we heed recent calls for data integration and synthesis in archaeology [109,110]. In doing so, we also build on previous studies that have identified reproducibility concerns in archaeology [111]. We first present our expert-sourced dataset designed to trace continental-scale patterns in stone toolkit structure, armature design and technological processes of blank production for the period from c. 15,000 to 11,000 years ago (cal BP). We then statistically assess similarities and differences both within and between the various cultural units in our dataset. Subsequently, we critically interrogate the efficacy of these cultural units in structuring lithic variability in the European Final Palaeolithic and earliest Mesolithic. Specifically, we evaluate (i) which groupings of traditionally named archaeological units can be recovered from the dataset based on different dimensions of lithic data; (ii) how coherent the various NACs are and what their discriminative power is; and (iii) to what extent we can trace patterns of cultural diversification over time.

## Data acquisition

**Fig 1:**
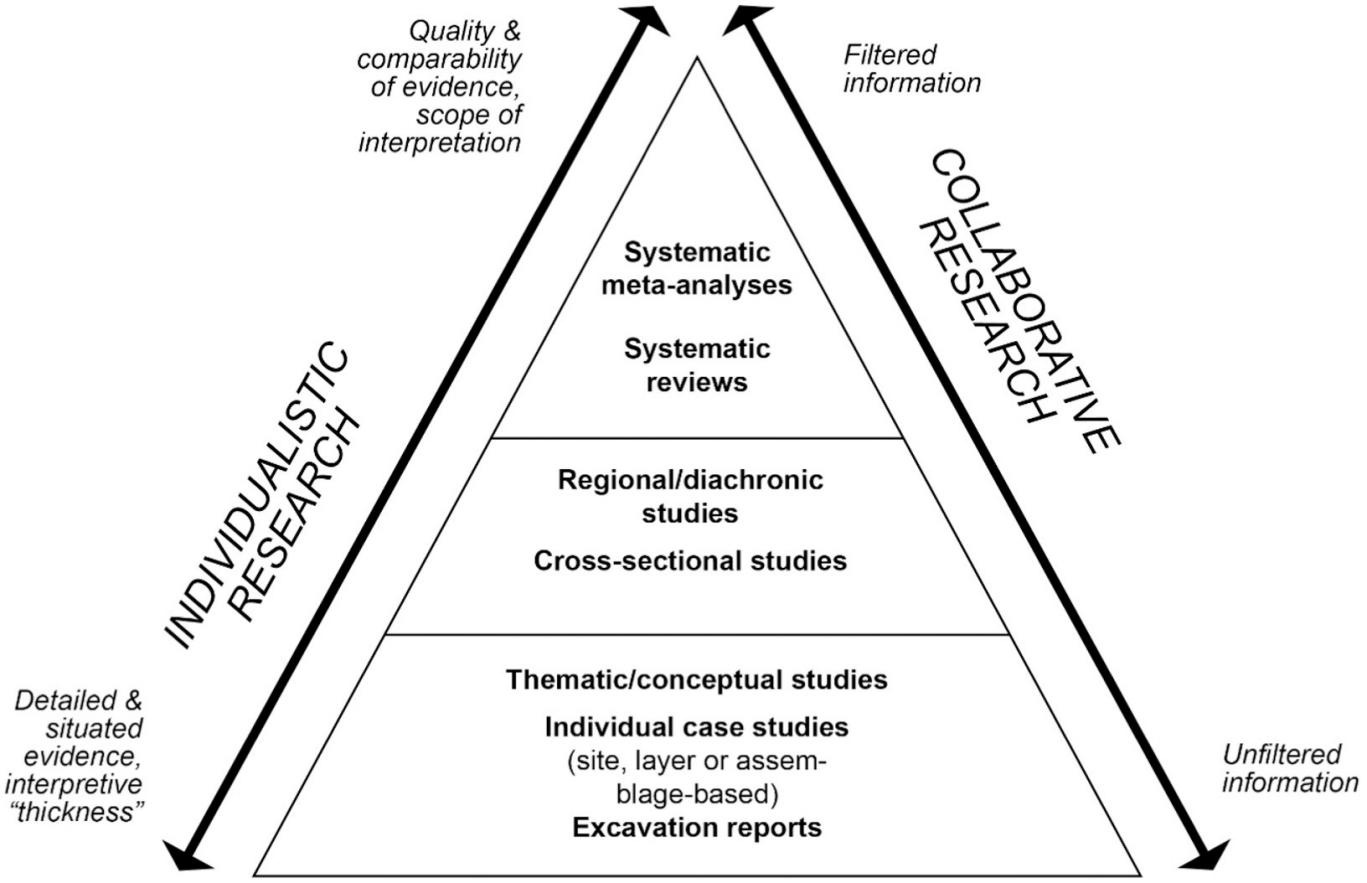
Pyramid of evidence in archaeological research and the place of higher-order analyses and collaborative practice. Modified from Hussain et al. [113].

**Fig 2:**
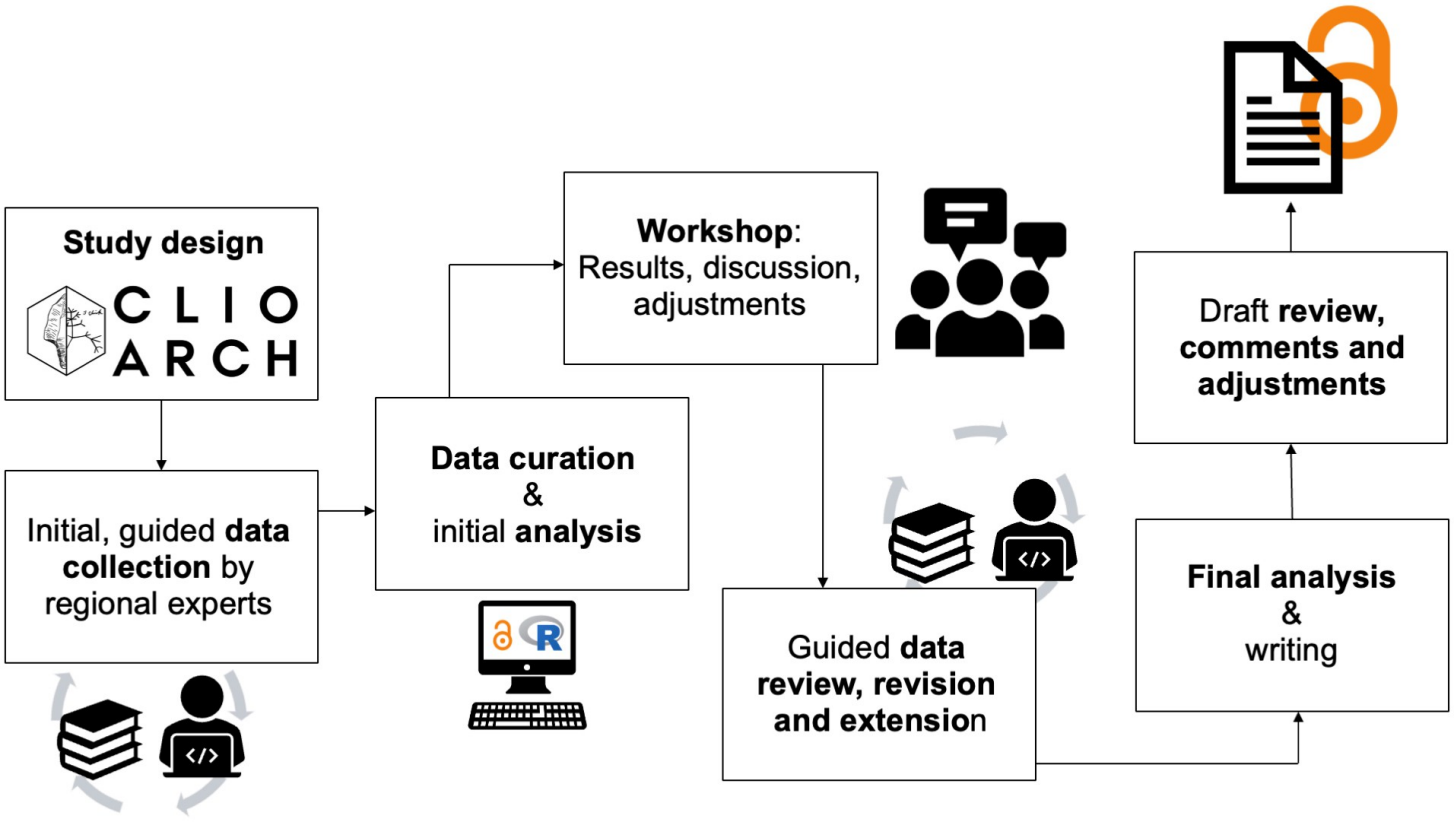
Flowchart of data-sourcing, analytical and interpretative process.

In the framework of the CLIOARCH project [112] and under the unfortunate shadow of the Covid pandemic, we used current communication and data-sharing possibilities to construct a novel integrated dataset on toolkit structure, retouched tool shapes and technologies associated with Final Palaeolithic/earliest Mesolithic foragers in Europe [113]. The purpose of this data collection was to facilitate syntheses and inter-regional synthesis (Fig 1). Unlike other disciplines such as the health sciences or ecology [114,115], comprehensive methodological guidelines or standards for archaeological meta-analysis and syntheses are yet to be established. We therefore devised a workflow that integrated first-hand empirical knowledge of regional specialists with the methodological expertise and theoretical framing of the lead team (Fig 2). Regional experts were sourced in the lead team’s network and had to have extensive first-hand experience with the particular area they represent; most commonly, these regional experts are also based in their respective region. Anchored in a series of virtual meetings and a workshop in which most team members participated, data collection occurred both before and after the workshop with room for revision with additional regional experts invited into the study as relevant. Data recording forms and guidelines were initially designed by the lead team and iteratively revised in dialogue with all contributors. The lithic data collected during this process are derived from published sources and each individual entry and object image can be traced to their respective sources. Subsequent analyses were conducted in the R computing environment [116], and the original raw data and code are available at https://doi.org/10.5281/zenodo.7940337.

## Materials and methods

### The database of named archaeological cultures

**Fig 3:**
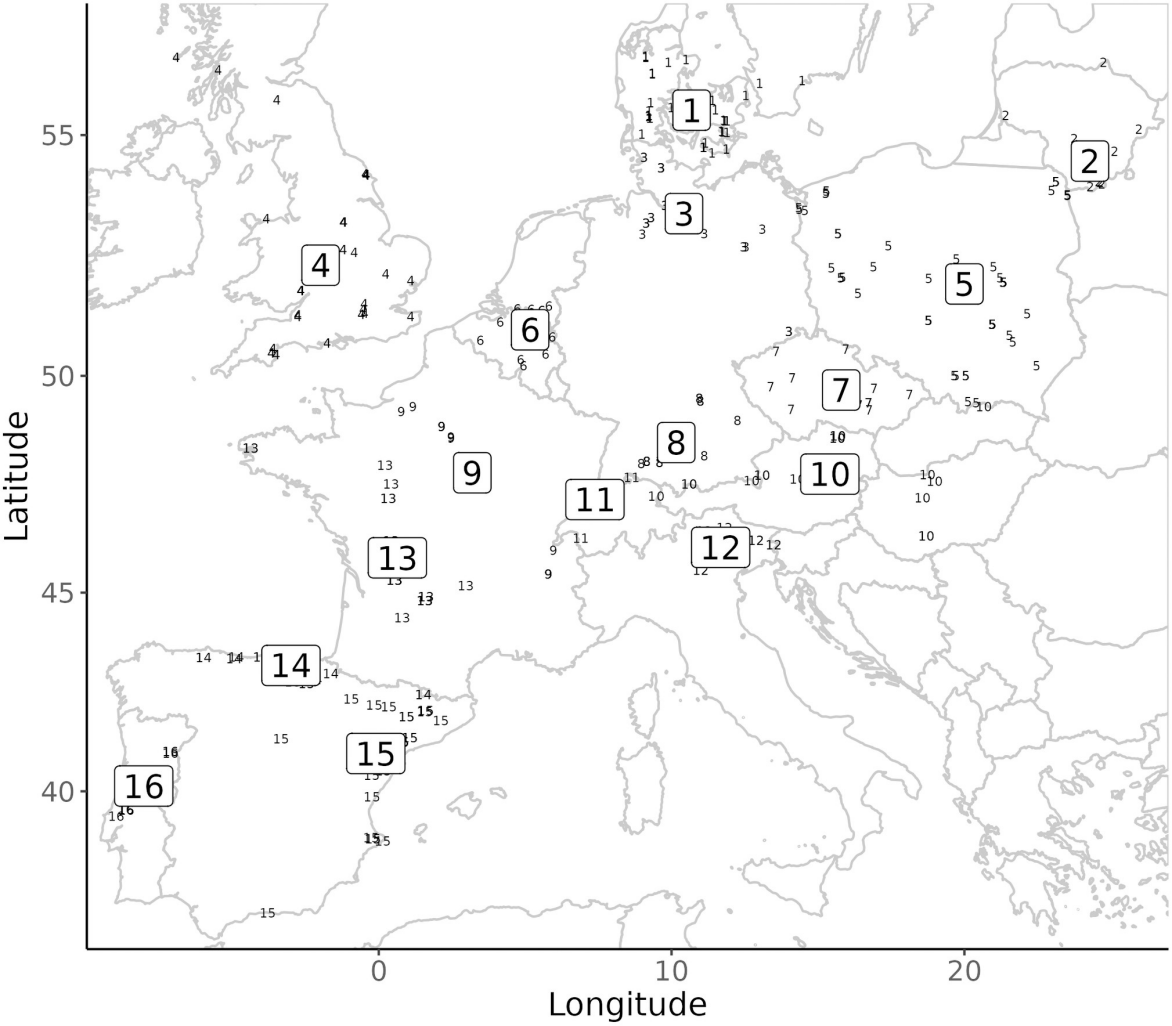
Regional units as used in this study (boxed large numbers) and the distribution of associated key sites (small numbers). Large numbers represent regional centroids (mean site coordinates). 1: Southern Scandinavia, 2: Lithuania, 3: Northern Germany, 4: Britain, 5: Poland, 6: Belgium and Southern Netherlands, 7: Bohemia and Moravia, 8: Southern Germany, 9: Northern France, 10: Austria, Slovakia, Hungary, 11: Switzerland, 12: Northern Italy, 13: Western France, 14: Cantabrian Spain, 15: Mediterranean Iberia, 16: Atlantic Iberia. A detailed breakdown of key sites can be found in **SI Table 2 in** S1 Data **and associated data**.

The dataset records a total of n = 86 named archaeological cultures (NACs) with associated lithic data derived from n = 350 key sites and their associated information on archaeological context (dating, site type, preservation) and integrity. These taxonomic units are placed within four millennial time-slices (TS) measured in calibrated years before present (BP = 1950; TS I: 15,000–14,000; TS II: 14,000–13,000; TS III: 13,000–12,000; TS IV: 12,000–11,000; Table 1) and cover a large portion of Northern, Central and Western Europe. The dataset is divided into n = 16 regions for the purpose of this study: Atlantic Iberia, Cantabrian Iberia, Mediterranean Iberia, Western France, Britain, Northern France, the Low Countries, Switzerland, Southern Germany, Northern Germany, Southern Scandinavia, North-eastern Italy, Austria/Slovakia/Hungary, Moravia/Bohemia, Poland, and Lithuania (Fig 3). These regional units act as heuristic ‘contextual areas’ [cf. 117,118] for our comparative technological, trait, and shape-based spatial analyses. Similarly, our temporal units defined by discrete millennia are—akin to artificial spits during excavation—thought to act as a metronome of cultural change not determined by climatic phases or chronozones to circumvent the so-called ‘modifiable reporting unit problem’ [119] of comparing units across a timeline, and so as not to pre-empt the question of environmentally driven culture change as implicated in taxonomic denominations and naming conventions.

**Table 1:** NACs submitted by regional experts, ordered according to time-slice (in cal BP) and latitude (see S1 Data, see S1 Table as well as S1 Fig in S1 Data for further details). ABP = Arch-backed point complex; CBP = curve-backed point complex; FBT = Flat Blade and Bladelet Technocomplex.

	Time-slices (ka BP)				
Region	I 15–14	II 14–13	III 13–12	IV 12–11	NACs/region
Southern Scandinavia	Havelte	Federmesser, Brommean	Brommean	Ahrensburgian, Maglemose	5
Lithuania		Hamburgian, Federmesser	Federmesser, Brommean, Ahrensburgian	Swiderian	5
Northern Germany	Hamburgian, Federmesser, Federmesser/ Brommean (?)	Federmesser, Havelte, Federmesser/Brommean(?), Brommean	Federmesser, Ahrensburgian	Maglemose	7
Britain	Creswellian/Late Magdalenian, Backed Blade/H-type CBP, Havelte	Backed Blade/H-type CBP, Havelte	Allerød CBP	Long Blade Industries/Epi-Ahrensburgian (FBT), Early Mesolithic	6
Poland	Magdalenian, Hamburgian	Kamienna variant, Witowian	Witowian, Brommean, Perstunian, Wolkuszian	Wolkuszian, Epi-Ahrensburgian, Swiderian, Swiderian-Ahrensburgian, Pludian	11
Belgium and Southern Netherlands	Magdalenian	Federmesser	Epi-Ahrensburgian	Epi-Ahrensburgian	3
Bohemia and Moravia	Magdalenian	Magdalenian, Epimagdalenian, ABP Federmesser, ABP Tishnovian	ABP Tishnovian	Mesolithic	5
Southern Germany	Late/Transitional Magdalenian	Late Palaeolithic	Late Palaeolithic, Atzenhofer group	Late Palaeolithic, Atzenhofer group, Beuronian A	4
Northern France	Early Azilian, Upper Magdalenian	Early Azilian, Late Azilian	Late Azilian	Belloisian	4
Austria, Slovakia and Hungary	Late Magdalenian, Late Epigravettian	Aziloid Alps, Aziloid—Tradition Eastern Central Europe	Aziloid Alps, Aziloid—Tradition Eastern Central Europe, Late Palaeolithic generic	Aziloid Alps, Epigravettiano recente, Aziloid—Tradition Eastern Central Europe, Late Palaeolithic generic	6
Switzerland	Late Magdalenian/ Technokomplex E, Older Azilian	Older Azilian, *Fazies Fürsteiner*	Older Azilian, *Fazies Fürsteiner*, Younger Azilian	*Fazies Fürsteiner*, Early Mesolithic I	5
Northern Italy	Late Epigravettian (Phase 2)	Late Epigravettian (Phase 2), Late Epigravettian (Phase 3)	Late Epigravettian (Phase 3)	Late Epigravettian (Phase 4)/Early Sauveterrian, Early Sauveterrian (Phase 4)	4
Western France	Early Azilian, Upper Magdalenian	Early Azilian, Late Azilian	Early Laborian, Late Azilian	Early Laborian, Late Laborian	5
Cantabrian Spain	Final Magdalenian, Azilian	Azilian	Azilian	Azilian	2
Mediterranean Iberia	Final Magdalenian, Upper Magdalenian	Epipaleolithic/ Epimagdalenian	Sauveterrian/ Sauveterroid Epipaleolithic, Epipaleolithic/ Epimagdalenian	Sauveterrian/ Sauveterroid Epipaleolithic	4
Atlantic Iberia	Upper Magdalenian	Final Magdalenian	Azilian	Azilian	3
**NACs/TS**	**17**	**24**	**23**	**26**	

Regional experts identified the corpus of cultural taxonomic units currently in use for the period between 15,000 and 11,000 cal BP in their assigned respective regions. This often includes chronological sub-units as indicated by prefixes such as ‘Early’, ‘Late’ or ‘Epi-’, as well as sub-units (‘groups’, ‘facies’) thought to derive from higher-order cultural taxa [cf. 120]. Following these traditional taxonomic assessments, this returns a total sample in which the existence/persistence of named cultures or groups per time-slice rises from 17 units in TS I to 24 in TS II, to then again fall to 23 in TS III, and to peak with 24 in TS IV (cf. SI Fig 1 in S1 Data for raw counts). For each NAC, experts were tasked with assembling a portfolio of key sites, exemplifying the lithic data on technology and toolkit composition, and from which individual artefact outlines and contextual information are sourced. Accordingly, each cultural taxonomic unit is anchored in a well-defined set of reference sites and their associated publications. Lithic toolkit composition and technological data are recorded on the scale of whole regionally bounded taxonomic units while lithic outline data can be traced to specific objects (Table 2).

**Table 2:** Overview of database modules and their composition and resolution. A comprehensive breakdown of all modules is presented in S3 and S4 Datas. Outlines of scrapers and borers/perforators have been collected but are not analysed in this paper. *The dataset comprises 86 unique named cultural taxa, two of which occur across two separate time-slice entries, totalling 88 distinct observations.

Module	Shorthand used in this paper	Sub-domains	Number of recorded traits	Data type	Total observations*	Scale
***Tool classes***	*Tools*	Armature/points; Domestic tools	24	Discrete	86 (88)	Named archaeological culture
***Organization of laminar technologies***	*Technology*	Blade-bladelet reduction strategies and core structure; Raw material economy; Blade structure and morpho-typology of laminar blanks; Other/non-laminar blank production systems and their interrelationships; Retouch tendencies (+ presence of microburin)	52	Discrete	86 (88)	Named archaeological culture
***Armature outlines***	*Outlines*	Armatures (AR)	-	Continuous	3512	Individual artefact
***Archaeological site metadata***	Metadata	Site context and data quality; Calculated site-quality scores; Register of processed and recorded tool outlines	25	Various	-	Site

Key sites were generally identified as those that hold rich information on lithic typology and technology, that are well-published (including lithic drawings and photographs), and whose chronostratigraphic position is relatively secure and reliable. However, not every key site in the database fulfils all these criteria, and some rather represent those mentioned most frequently in the relevant body of literature. For each site included in the database, a quality score was calculated based on selected contextual data entries (SI Fig 2a and 2b in S2 Data). The summed quality score (0–6) reflects chronological reliability, stratigraphic resolution, assemblage integrity and coherence as well as when a given site was investigated. A detailed explanation of the scoring procedure including all relevant entries is given in S2 Data.

The lithic database is subdivided into four data modules, three of which contain information on different aspects of lithic technology and toolkit structure, while the fourth records contextual and locational information of each key site (cf. Table 2). The first module contains presence/absence data on 24 retouched tool classes, split into 14 armature classes and 10 domestic tool classes. The second module encompasses information on the organization of laminar technology, raw material economy and retouch patterns, broken down into 52 individual techno-typological traits grouped into five sub-categories. The third module gathers 3512 lithic armature outlines compiled from the literature and other available image sources such as photographs. The artefact classes included in this latter category are points and backed implements including (presumed) projectile elements. These reflect the artefact classes most used to construct cultural taxonomic groups. Experts were encouraged to submit >20 images from the relevant key sites, although the total number of available images per cultural taxon varies by data availability and publication status. Notably, for some units it proved remarkably difficult to assemble even moderately sized image datasets. The focus on objects held to be diagnostic for each unit also makes our analysis conservative in relation to the likely variability represented by artefacts not drawn or photographed. In total, the database comprises 88 individual NAC observations each associated with 24 discrete tool class variables and 52 discrete technological variables. A complete register of data modules, artefact classes, traits and other analytical variables is provided in S3 and S4 Datas. In addition, each of the four data modules is shortly described in separate subsections below.

### Toolkit composition data

To facilitate broad-scale, cross-regional comparison while accounting for the particularities of European Final Palaeolithic/earliest Mesolithic retouched tool assemblages, emphasis is here placed on lithic points and purported projectile implements. These tool forms are considered indicative of cultural change, and they traditionally play a privileged role in the construction of cultural taxonomies. Commonly referred to as artefactual index fossils they are considered diagnostic in ways similar to type fossils in palaeontology, from where the notion was borrowed in the nascent years of Palaeolithic archaeology [121]. Unretouched components are not considered here. The full list of the n = 24 retouched tool classes including their formal definitions is provided in Supplementary Information S3.2 in S3 Data. We regard these classes as morphotypes within the available design space. Tool classes are generally consistent with established definitions and taxonomies [10,61,122–125] but foreground nodal differences in tool shape and morphology, sometimes with the addition of size.

Within the 14 retouched tool classes catalogued as ‘armatures’ [following broader definitions by 1,126–129], the main distinctions are made between simple, tanged, and shouldered points, backed implements (including arch-backed and angle-backed variants), and different geometric forms such as triangles and trapezes. The ‘domestic’ tool category (*outils du fonds commun*) includes ten tool classes, encompassing endscrapers, burins, borers (‘becs’ and other borer-like forms) and larger unretouched blade knives which have recently been recognized through micro-wear studies conducted on an as yet limited number of assemblages from only a few regions as an important tool variant in the European Final Palaeolithic and earliest Mesolithic [69,74,130–134]. Tool classes are recorded as discrete presence/absence, or ‘n/a’ in case of insufficient information, at the level of each NAC. Presence data indicates the consistent occurrence of the respective tool morphotypes within a given taxonomic unit.

### Technological data

The 52 recorded technological traits also capture taxa-level presence/absence data, with presences reflecting taxa-wide tendencies and recurrences at regional scales. All individual traits are listed and specified in Supplementary Information S3.3 in S3 Data. The lithic technology module is organized in five sub-domains designed to capture different types of lithic information for each observation:

1. A total of 26 traits describes *laminar reduction strategies* and *core organization/structure*, cataloguing information on the number of separate reduction strategies, the directionality of blank production, the configuration and geometry of exploitation surfaces, the type and extent of core preparation and the mode of knapping.
2. *Raw material economy* is described by five traits relating to the diversity of worked and transformed lithic raw materials and the degree of raw material adaptation and specialization.
3. The sub-domain *laminar blanks*, with ten traits, addresses the structure, layout and technological characteristics of blades including outline shape, profile configuration, and type of platform preparation.
4. Seven traits record the presence and interrelationship of *other production goals*, especially flake and bladelet production but also the relationship between blank and tool production more generally.
5. Four traits capture information on the character of *blank modification* across tool assemblages and records the systematic presence of lithic products associated with the *microburin technique*.

### Outline data

Tool outline data were collected from published or otherwise available image sources (lithic artefact drawings and photographs) on the level of individual key sites, with the aim of assessing the efficacy of these tool forms in discriminating specific cultural taxa and their relations. While the outlines collected here still only describe a limited subsection of all tools found at even this already selected sample of sites—most likely the most regular and complete ones selected for drawing or photography [cf. 135,136] –they facilitate an assessment of the variability of tool forms beyond purported type diagnostics. Outlines were extracted, following the protocol developed by Matzig [137], from complete artefacts or from artefacts for which the missing parts could reliably be reconstructed or have been reconstructed in the original publications.

The *Outline* dataset includes n = 3512 digitized outlines of retouched stone tools falling into the functional category ‘armatures’. These armatures comprise all formal tool classes included in the corresponding category found in the tool class module of the database (cf. Supplementary Information S3.2 in S3 Data). A full overview of all outline database is given in S4 Data.

Notably, the availability of artefact images differs markedly across regions and time-slices. This is the consequence of practical scholarly constraints—resources for drawing and photography—and differences in research traditions and publication cultures, which sometimes assign differential value to imaging and drawing [138]. In some cases, this also reflects reporting biases (the selection of what artefacts to report at all) and representation biases (which artefacts to draw or photograph in a given research context).

### Metadata: Site context and data quality

The fourth module of the database encompasses information such as geographic location (latitude/longitude), dating information, as well as background knowledge on the archaeological context of the assemblages in question and their relative quality and reliability. Contextual data include details on the type of archaeological site, stratification and geological position of lithic assemblages, the coherence of these assemblages, and the preservation of faunal remains. The availability of lithic outlines for each site and time-slice is also noted here. The complete list of site-specific contextual information is provided in Supplementary Information S3.4 in S3 Data.

## Methods

We here explore the extent to which data-driven computational approaches to the presently available lithic evidence are capable to recover patterns of similarity that align with NAC’s semantic and chronological affinities. Our goal is to assess the compositional integrity of expert-proposed NACs and to examine and quantify the consistency of conventional naming practices in European Terminal Pleistocene and incipient Holocene archaeology. We also measure the diversity of lithic forms as reflected in outline shapes of armatures in order to examine processes of cultural diversification across the Pleistocene-Holocene transition.

### Data preparation and analysis

#### Trait and image data

*Trait data*. In the datasheet for *Tools* and *Technology* each row contains information on a single NAC. The columns each describe a different character trait (see above and S3 Data for a complete breakdown of traits), each of which can either assume the states absent (0), present (1), or not available (n/a). The last category reflects instances in which expert editors could not determine the appropriate character state due to regionally specific research histories, traditions, and uneven research priorities, or the nature of the published or otherwise available information. Based on the resulting discrete dataset, we then calculated the pairwise distances between individual regional NACs using the Gower distance measure provided by the function *vegdist()* of the R package *vegan* [139]. This process returns similarity matrices for toolkit composition and lithic technological organization respectively.

*Outlines*. We extracted the (continuous) outline data from available photographs and drawings of lithic armatures by using the R package *outlineR* [137], following the workflow described in Matzig [140], Matzig et al. [141], and Araujo et al. [142]. Using the *Momocs* package [143], all obtained outlines were smoothed by a ‘moving average’ procedure to avoid digitization noise, and then interpolated to yield 500 semi-landmarks, with the starting landmark placed at the distal extremity (typically the tip) of its corresponding artefact.

Deploying *Momocs*, we submitted the resulting dataset to an Elliptic Fourier Transformation (Elliptic Fourier Analysis; EFA) and retained the number of harmonics that describe 99.9% of the harmonic power. We then conducted a principal components analysis (PCA) on these harmonics. We use all 68 resulting principal component (PC) scores to calculate the pairwise Euclidean distance between each outline by employing the base R function *dist()*. In analyses concerning the comparison between discrete time-slices, observations of all three domains that span several time-slices had to be discretized and duplicated to appear in each of the time-slices they encompass.

#### Testing the taxonomic units

To assess similarities and differences between individual NACs across different lithic data domains and to compare these with taxonomic denominations and naming practices, we subject the dataset to different complementary quantitative analyses. We (i) deploy cluster analysis to assess the overall structure in our dataset and (ii) use the Mantel test to assess the relevance of time and space as structuring factors. The former allows for effective visual inspection and assessment of between-NAC distances. To more systematically and directly compare similarities and differences implied by Final Palaeolithic/earliest Mesolithic naming practices, we additionally group individual NACs into seven higher-order macro-units based on semantic affinity and relatedness of the expert-submitted taxonomic units (Table 3). Only two NACs—both designated as generic Late Palaeolithic—were not assigned to a macro-unit. These macro-units are created as a heuristic means to examine within- and between-group differences among NACs based on pairwise dissimilarity measures and so to facilitate the assessment of compositional integrity and coherence. The creation of such higher-order units is not trivial, and the proposed organization is a compromise between naming similarities and current discussions on the overall placements of NACs within the European Final Palaeolithic/earliest Mesolithic. The macro-units used here thereby seek to take implied chronological and culture-historical relationships into account. Chronological relatedness is expressed, for example, in prefixes such as Epi-, Late, or Early, while suffixes such as -oid indicate derivation, origin or similarity, and specific geographic tags or the naming of particular (idiosyncratic) cultures or unique groups is taken as the semantic signature of distinctiveness.

**Table 3:** Assignment of individual NACs into seven heuristic higher-order macro-units to facilitate the analysis of compositional integrity and diversity.

Higher-order macro-units	NACs included	N_NAC_ subsumed
***Magdalenian*** *sensu lato*	Late Magdalenian Magdalenian Epimagdalenian Late/Transitional Magdalenian Late Magdalenian/Technokomplex E Upper Magdalenian Final Magdalenian Epipaleolithic/Epimagdalenian Hamburgian Havelte Kamienna variant Creswellian/Late Magdalenian	24
***Epigravettian*** *sensu lato*	Late Epigravettian Epigravettiano recente Late Epigravettian (Phase2) Late Epigravettian (Phase3) Late Epigravettian (Phase4)/Early Sauveterrian	5
*Arch-back point (ABP) complex*: ***Azilian***	Aziloid—Tradition Eastern Central Europe Aziloid Alps—Tradition Southern Germany Older Azilian Younger Azilian Early Azilian Late Azilian Azilian (Pyrenees) Azilian	16
*Arch-back point (ABP) complex*: ***Federmessergruppen***	Federmesser ABP Federmesser ABP Tishnovian Fazies Fürsteiner Atzenhofer group Witowian Backed Blade/H-type CBP Allerød CBP	11
***Flat Blade and Bladelet Technocomplex/Long-Blade Industries*** *(FBT/LBI)*	Epi-Ahrensburgian Early Laborian Late Laborian Belloisian Long Blade Industries/Epi-Ahrensburgian (FBBT)	6
***Tanged-point complex*** *(TPC)*	Bromme Brommean Perstunian Ahrensburgian Swiderian Swiderian-Ahrensburgian Pludian Wołkuszian	14
***Mesolithic***	Early Sauveterrian (Phase 4) Early Sauveterrian Sauveterrian/Sauveterroid Epipalaeolithic Mesolithic Beuronian A Maglemose Early Mesolithic Early Mesolithic I	9
*Not assigned*	Late Palaeolithic Late Palaeolithic generic	2

The seven macro-units assembled in this way (see Table 3) allow us to test for differences among NACs currently discussed in the literature, such as between a larger sphere of Magdalenian- and Epigravettian-affiliated NACs and between Azilian and Federmesser-related units. We also seek to test the significance and replicability of the recently proposed FBT complex as well as its relationship to NACs belonging to the penecontemporaneous Tanged Point Complex (TPC) and the Mesolithic. The Mesolithic macro-unit is relatively heterogeneous in terms of its constituent NAC labels, including all units in the dataset traditionally addressed as earlier Mesolithic (e.g. Maglemose, Beuronian and Sauvetterian) and all generic Mesolithic designations dated to the Pleistocene/Holocene transition or the earliest part of the Holocene. We can interrogate the relative homogeneity of NACs grouped as ‘Mesolithic’ and ask whether this generic ‘Mesolithic’ is better defined than the Magdalenian or the ABP/Azilian macro-units respectively. Based on the character of the individually named units contained within each macro-unit, we can further devise testable hypotheses as to the expected within-group coherence of these units. While macro-units such as the Magdalenian, Epigravettian and ABP/Azilian are anticipated to return high degrees of within-group coherence, macro-units such as TPC, FBT/LBI and the Mesolithic are expected to be less well-defined. Additional expectations vis-à-vis the structure of variability encompassed by the seven macro-units can be brought to the table, especially with regard to different lithic data domains: we may for example anticipate that ABP/Azilian and TPC are relatively well-defined in terms of toolkit compositions whereas macro-units such as FBT/LBI and to some extent Magdalenian and Epigravettian show higher levels of technological consistency. Definitions of these NACs between our study regions through detailed technological research has not received equal attention everywhere. We here seek to quantify and assess the relative performance of *Toolkit* and *Technology* data in meeting these expectations.

Finally, this higher-order macro-unit framework also allows us to computationally assess the relative importance of different traits in our dataset to define semantic and culture-historical affinities contained in the recorded NACs. Using a reductive machine learning classification approach, we systematically examine the intuition that different NACs are defined on different criteria and further qualify the trait space that matters most for the description of overall lithic variability in the timeframe of our investigation. In doing so, the lithic data at hand, especially the trait data, offers advantages and disadvantages. The data are coarse-grained and somewhat simplified for the purpose of the present analysis. They clearly reduce the relational complexity of lithic technical systems and should therefore not be expected to provide a detailed, fine-scale resolution of the issues at hand. By the same token, however, our high-level approach has the advantage of potentially identifying broad-scale structure-giving characteristics otherwise lost in the thickness of observational detail.

#### Testing for unit coherence using distance matrices

Our approach for testing the integrity of Terminal Pleistocene/earliest Holocene NACs is based on the determination of pairwise Gower distances for the *Tool* and *Technology* datasets respectively, and the Euclidean pairwise distances derived from the PCA scores of the *Outline* dataset described above. We reason that if the higher-order classes are coherent and have discriminative power regarding the total variability space, the pairwise distances of NACs within higher-order grouping should be smaller than the pairwise distances of the same NACs of that higher-order grouping to other randomly selected NACs.

Using this dataset, we calculated the standardised effect size (SES), comparing the observed distance within a given group to the distance expected under null-distribution conditions. Calculating SES requires normality of the null-distribution [144]; as we sample the pairwise observations 10.000 times for the *Tools* and *Technology* data, and 100.000 for the *Outlines* data, we assume that the central limit theorem, and thus null normality, is in place:

$${SES}_{metric}=\frac{{Metric}_{observed}-mean\left({Metric}_{mull}\right)}{sd\left({Metric}_{null}\right)}$$

**Fig 4:**
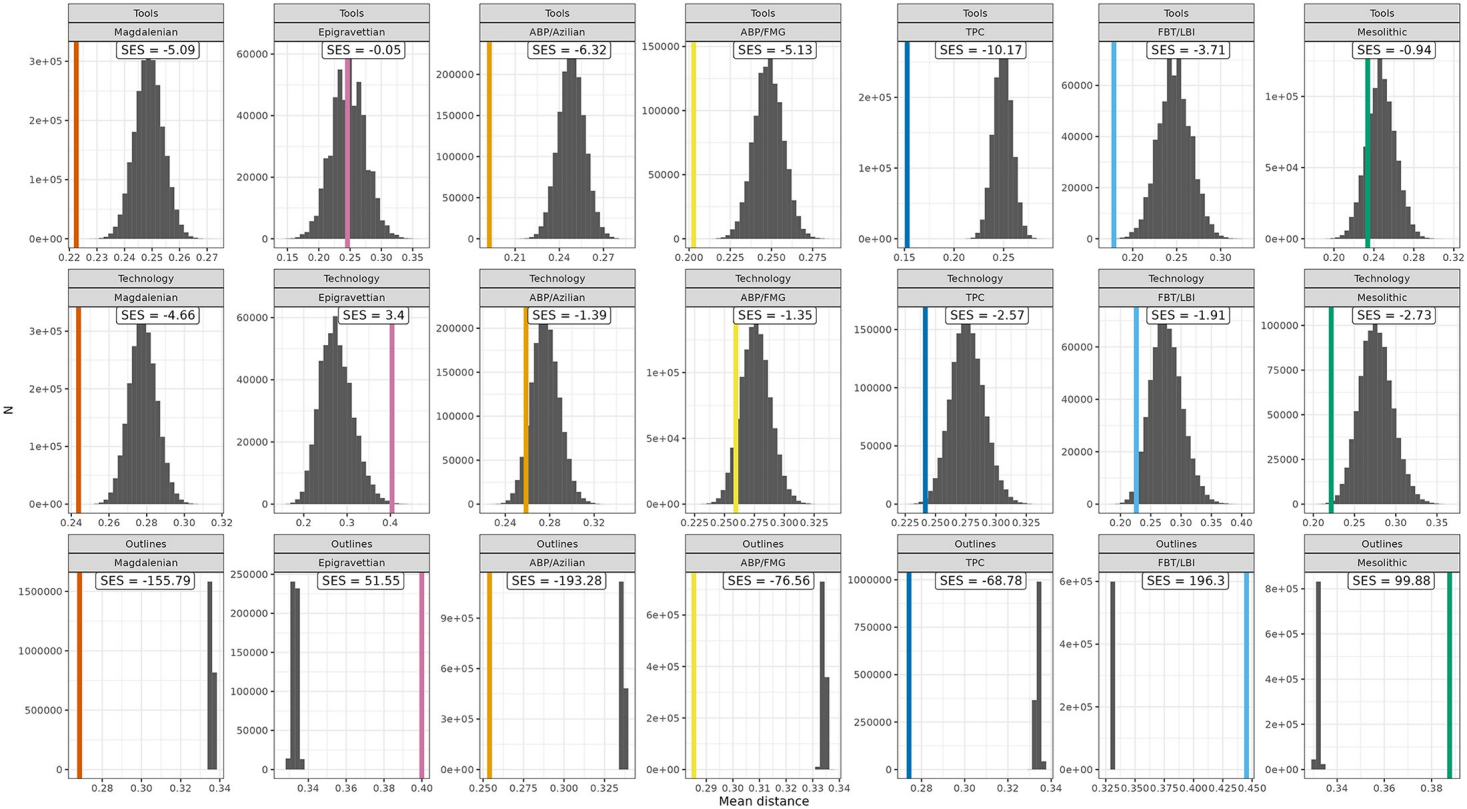
Within-group mean distances (coloured) of higher-order macro-units and the mean distances of the associated null-distributions (grey bars) for each data domain. The SES-value informs about the validity of the higher-order groupings. SES-values between -2 and 2 (-2>SES<2) suggest that the mean distance within a given higher-order grouping is not significantly different from the mean distance of groupings consisting of randomly drawn NACs. SES-values of -2 or lower (SES≤-2) instead suggest that the observations within a higher-order grouping are significantly more similar to one another than if they had been grouped together by chance. The opposite is the case for SES-values of two or more (SES≥2), pointing to substantial internal variability. In general, the further the coloured line is away from the randomized distance measures obtained (grey bars) the better does the proposed classification perform. The difference in SES values between the Outlines domain on the one hand and the Technology and Tools domains on the other is driven by sample size.

SES values indicate whether the within-group distance is larger or smaller than expected by chance. SES is negative when the observations within the group are more similar than in a random distribution. If SES is positive, accordingly, observations within a taxonomic group are less similar than between randomly distributed NACs. Results are significant if SES values are <-2 or >2, indicating that the random difference is two-times larger than the corresponding standard deviation. A SES value of 2/-2 approximates the 5% significance level [145; Fig 4].

**Fig 5:**
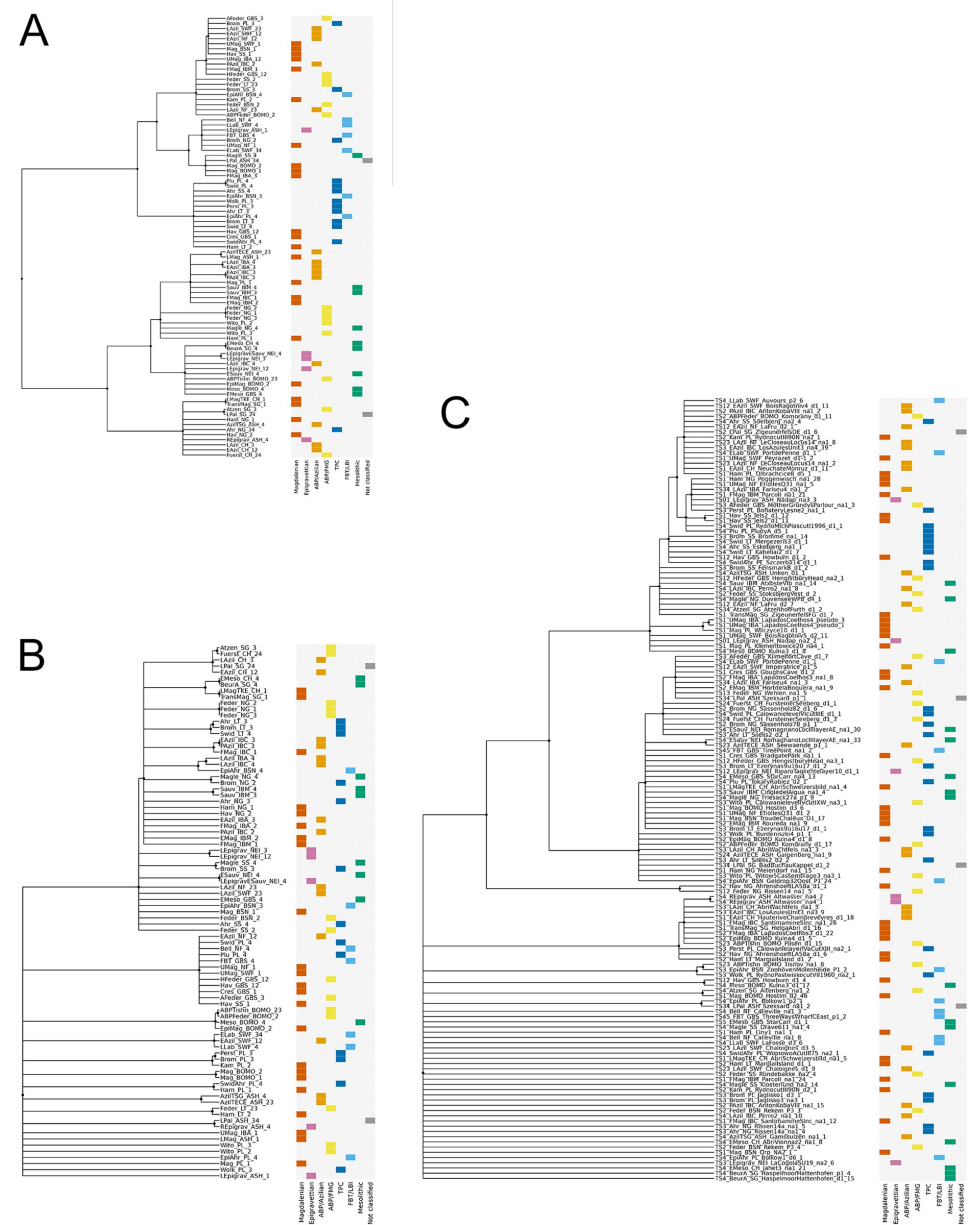
**A.** Bootstrapped dendrogram of Tools using Ward’s [147] method based on the Gower distance, conducted at the level of named archaeological cultures (NACs). Clades below a threshold of 50% were collapsed. The coloured grids show the tips’ associations with higher-order macro-units. **B**. Bootstrapped dendrogram of Technology using Ward’s [147] method based on the Gower distance, conducted at the level of named archaeological cultures (NACs). Clades below a threshold of 50% were collapsed. The coloured grid shows the tips’ associations with higher-order macro-units. **C.** Bootstrapped dendrogram of Outlines using Ward’s [147] method based on the Euclidean Distance of the obtained PCA data obtained, conducted at the level of individual sites. Clades below a threshold of 50% were collapsed. The coloured grid shows the tips’ associations with higher-order macro-units. A stratified subset has been drawn to include two random outlines for each individual NAC.

As an alternative way to visualise and explore the similarities between NACs within the domains and their hierarchical organisation, we plotted the pairwise distance matrices in dendrograms using Ward’s method [cf. 146,147] for the *Tool*, *Technology* and *Outline* domains. Since the number of armature outlines is so high, we subsampled these when constructing the dendrogram. The subsampling was conducted in a stratified way using the *splitstackshape* R package [148], where we chose two outlines per NAC whenever available. The dendrograms were bootstrapped 1000 times, using the R package *ape* [149]. The resulting consensus trees retain all branches at a bootstrap value ≥ 50%. Branches with a bootstrap value below that threshold were collapsed (Fig 5).

#### Comparing structural inter-domain consistency using tanglegrams

Toolkit-technology relationships are often considered important in long-term lithic evolution, and much traditional work assumes that blank production technologies and toolkit organization strongly influence each other, and thus co-vary. To assess this hypothesis, we juxtapose the hierarchical structure of variation contained in the *Tools* and *Technology* datasets and the respectively constructed dendrograms for shared organizational and associative properties regarding individual NACs and our higher-order units. A tanglegram approach was originally developed to investigate co-evolutionary dynamics across associated systems (e.g. hosts and parasites); it allows direct visual inspection and ready comparison of independently derived trees or networks [150]. The tanglegram (Fig 7) used in this study is constructed by deploying the *dendextend* package [151] and utilizes the *Tools* and *Technology* dendrograms described above as input.

#### Using CART to determine key definitional traits for higher-order groupings

We deploy Classification and Regression Trees for Machine Learning [CART; 152] to assess the relevance of individual *Tools* and *Technology* traits to discriminate between and define our seven higher-order archaeological macro-units, and to in this way identify the most significant variables organizing the larger NAC-dataset. CART uses the trait data recorded for all individual NACs assigned to each macro-unit in order to make predictions about a target outcome variable, in our case the hypothesized taxonomic macro-units. This analysis asks which of the traits and their character states (0 or 1) best predict a given macro-unit. CART calculates a binary decision tree representing the trait-choices underpinning and explaining the hypothesized macro-unit structure of the two datasets. CART was performed with ‘Partial data’ and ‘Minimum error’ pruning in *DisplayR*. Detailed CART results are provided in SI Tables 4 and 5 in S4 and S5 Datas.

#### Testing for the influence of geography and time using Mantel tests

The Mantel test [153] is a common even if criticized [154] approach to test for correlation between two distance matrices. Originally designed for time-series analysis, the test is today commonly deployed in population genetics to evaluate the overall influence of spatial distance on genetic dissimilarity [155]. An extension of the Mantel test is the Mantel correlogram, which is suited to study non-linear relationships such as—in our case—between cultural distance and geographic distance intervals across space. In a Mantel correlogram, the distance matrices are divided into sub-matrices based on non-overlapping ranges of geographic distance. The correlogram then represents and visualises the Mantel correlations between each (cultural) distance sub-matrix and the mid-point of its associated geographic range sub-matrix [see 156]. We conducted Mantel tests as well as Mantel correlogram analysis using the R package *vegan* [139] on all three datasets. For the *Tools* and *Technology* datasets the pairwise Gower distances were used, and the Euclidean distances for the *Outlines* dataset, which were tested against their respective geographical distance matrices. The geographical distances were calculated based on the haversine distance measure employing the *distm()* function drawn from the *geosphere* package [157]. Distances for *Tools* and *Technology* were calculated between the regional site-centroids of individual NACs (cf. Fig 3). Distances within the *Outlines* dataset were measured for the geographically explicit coordinates associated with individual key sites. To test the relationship between cultural and geographic distance, we correlated cultural distance with distance in time using Spearman’s rank correlation coefficient [cf. 158]. We subset all three data sets (*Outlines*, *Tools*, and *Technology*) to observations which span only a single time-slice and use the Euclidean distance between time-slices as proxy for chronological distance. The resulting Mantel correlograms’ p-values are corrected for multiple testing using the Hochberg [159] method.

#### Testing for chronological change in artefact shape variation

To examine diachronic change in the 2D shape variation of lithic armature (AR), we calculated outline disparity within each of the four discrete time-slices (I-IV) using all outlines in the dataset. Disparity is defined here as the amount of total morphological variation, measured as the sum of variances within the outline PCA data of each time-slice. To determine the disparity values for each time-slice, we made use of the *dispRity* package [160]. Following Matzig and colleagues [141], we use disparity measures as a multivariate summary statistic of overall artefact-level variation akin to univariate measures such as the Coefficient of Variation (CV). The latter has previously been used to assess diversification and, by inference, cultural transmission dynamics in material culture [161,162]. Together with the quantitative evaluation of the degree to which cultural taxonomic variation is structured in the three datasets, armature disparity measures derived from the *Outlines* dataset provide complementary insights into diachronic design space dynamics within particular artefact classes, and thus offer a new perspective on processes of morphological diversification, and possibly normativity and cultural transmission, in lithic tool domains, especially presumable components of armatures, which are thought to track cultural developments particularly well at this time [45].

## Results

Beginning with the Standardised Effect Size measure, several interesting dimensions of discriminability in relation to the major cultural taxonomic units in our dataset are revealed (Table 4). These results show that macro-unit coherence is structured differently across data domains and that different similarity expectations can be varyingly recovered, underlining the complexity of NAC interrelationships. The Magdalenian, ABP/Azilian, ABP/FMG, TPC and, interestingly, FBT/LBI return significant SES values below -2 in toolkit structure (*Tools*), showing their general discriminability in this data domain. In the *Technology* dataset, Magdalenian, TPC and Mesolithic display strong within-group similarity, while the Epigravettian macro-unit produces SES values above 2, indicating notable within-group variability in laminar technological organization. This may be the result of the limited temporal and spatial representation of the long-lived Epigravettian within our dataset, considering that recent technological studies have identified considerable diversity in the maintenance strategies of retouched tools [e.g. 48,163]. Armature shapes (*Outlines*) reveal significant within-group similarity in the Magdalenian, ABP/Azilian, ABP/FMG and TPC macro-units, while the Epigravettian, FBT/LBI and Mesolithic yield SES values consistent with pronounced within-group 2D-shape variability. Magdalenian and TPC show significant within-group coherence across all three data domains, rendering them the best-defined macro-units within the dataset.

**Table 4:** Results of the distance-based significance tests of class attribution. Standardised Effect Sizes (SES) between -2 and +2 correspond to non-significant differences. SES results ≤-2 (bolded values) indicate that the observed difference is larger than two times the standard deviation. These latter groupings are thus significantly aggregated and more similar than random NAC associations in the sample. SES values of ≥2 (italicised values) suggest the opposite relationship, suggesting substantial within-group variability. Macro-units associated with SES values greater than -2/+2 are marked with *.

Data domain	Higher-order unit	N_NAC_ in higher-order grouping	Standardised Effect Size
**Tools**	*Not classified*	2	0.28
	Mesolithic	10	-0.94
	Magdalenian*	24	**-5.09**
	TPC*	14	**-10.17**
	FBT/LBI*	7	**-3.71**
	Epigravettian	7	-0.05
	ABP/Azilian*	18	**-6.32**
	ABP/FMG*	14	**-5.13**
**Technology**	*Not classified*	2	*2*.*37*
	Mesolithic*	10	**-2.73**
	Magdalenian*	24	**-4.66**
	TPC*	14	**-2.57**
	FBT/LBI	7	-1.91
	Epigravettian*	7	*3*.*4*
	ABP/Azilian	18	-1.39
	ABP/FMG	14	-1.35
**Outlines**	*Not classified*	27	-0.21
	Mesolithic*	537	*99*.*88*
	Magdalenian*	691	**-155.79**
	TPC*	347	**-68.78**
	FBT/LBI*	508	*196*.*3*
	Epigravettian*	233	*51*.*55*
	ABP/Azilian*	698	**-193.28**
	ABP/FMG*	471	**-76.56**

Interestingly, other macro-units are well defined in one data domain while showing pronounced variability in another. These disjunctions are particularly notable in the *Tools*-*Technology* relationships. Toolkit structures within the Mesolithic macro-unit are not significantly different from other random within-sample NAC associations, but technological traits suggest strong discriminability. This is consistent with the hypothesis of increased toolkit diversification in the Mesolithic *sensu lato* and that blanks were selectively transformed into specific retouched morphotypes depending on varying ecological and cultural constraints and/or motivations. The Arched-backed macro-units (ABP/Azilian and ABP/FMG) perform consistently well regarding *Tools* and *Outlines* similarity, as expected given the importance of diagnostic tool forms in their recognition, but do not show within-group *Technology* similarities that are significantly greater than random NAC associations within the dataset. Interestingly, macro-units that span the Pleistocene-Holocene boundary such as FBT/LBI, Epigravettian and the Mesolithic are characterised by substantial within-group variability in armature shapes (*Outlines*), consistent with long-standing proposals that tool shape differentiation becomes increasingly important at the transition to the Holocene across the continent. These results are encouraging but also show that NAC naming practices are currently underwritten by different definitional criteria and data.

Fig 4 shows in greater detail how the group assignments perform *vis-a-vis* randomized NAC-comparisons across all three data domains. With the notable exception of the Mesolithic, all macro-units are better defined by toolkit structure (*Tools*) than techno-economic organization (*Technology*). The Magdalenian is consistently well-defined across all data domains and ABP/Azilian, ABP/FMG and TPC produce notably similar performance patterns, with strong discriminability regarding *Tools* and *Outlines* and slightly reduced discriminability in the *Technology* domain. Similarly, Epigravettian and FBT/LBI are characterised by broadly comparable performance signatures with decreasing discriminability from *Tools* via *Technology* to *Outlines*. This confirms the chronological pattern outlined before, although the pronounced within-group variability of tool shapes (*Outlines*) in the macro-units dated to the end of the study period may be the result of different processes. On the one hand, tool production becomes increasingly separated from blank production, exemplified by whole-shape altering retouch strategies heralded by geometric forms. On the other hand, the use of unretouched products as tools not captured in the dataset may produce a biased picture—a possibility that is especially relevant for the FBT/LBI where large unretouched blade-knives have been identified as an important production goal. This would be broadly consistent with current understandings of the FBT/LBI complex as defined primarily in terms of its particular laminar blank production and core volume management technology [cf. 71,74]. That said, SES values for FBTI/LBI *Technology* only narrowly miss the significance mark (-1.91) and toolkit structure (*Tools*) turns out to be a hitherto underestimated defining factor (-3.71).

Fig 5A–5C capture the hierarchical structure within the three datasets as bootstrapped dendrograms, where the *Outlines* dendrogram is built on a stratified subsample of the total armature dataset to mitigate sample size unevenness across NACs and higher-order groupings. Overall, the topologies of these trees differ across data domains. A deep hierarchical structure, in which NACs that are semantically alike are sorted into discrete clusters is not consistently evident. At the same time, NACs occurring in the same regions and submitted by the same expert often tend to cluster together, which suggests either strong filtering by analysts or more pronounced regionality than typically assumed. The toolkit dendrogram (Fig 5A), in line with the results reported above, appears to be better structured than the *Technology* and *Outlines* dendrograms. The *Tools* dendrogram reveals two larger superclusters which broadly separate the Mesolithic and Epigravettian from TPC and FBT/LBI. TPC-associated NACs cluster particularly well and tend to occupy different clusters than FBT/LBI within the same supercluster. This separation would be even clearer if NACs designated as Epi-Ahrensburgian from the Low Countries—the assignment of which to either the Ahrensburgian or the Epi-Ahrensburgian is debated—would have been classified as TPC rather than FBT/LBI, suggesting that the Epi-Ahrensburgian is more similar to TPC-related NACs on the level of *Tools*. This may be taken to confirm that beginning in TS III, there is a tendency of increasing toolkit differentiation in these macro-units. Interestingly, northern Bromme instances (Northern Germany, Southern Scandinavia and Poland) cluster outside the main TPC cluster and closer together with FBT/LBI but also ABP/Federmesser and ABP/Azilian instances attributed to the upper supercluster, suggesting that the Bromme and associated large tanged points in the Terminal Pleistocene are closely associated with the ABP complexes rather than other groups characterised by tanged points [see 95,164].

Notably, the separation between Mesolithic and Epigravettian on the one hand and TPC and FBT/LBI on the other broadly describes a north-south division. It is interesting in this regard that the Southern Scandinavian Maglemose is associated with the supercluster containing most northern TPC and FBT/LBI-associated NACs. Conversely, the Epigravettian from the microregion comprising Austria, Slovakia and Hungary clusters neatly with FBT/LBI. The Ahrensburgian of Northern Germany is also not associated with other TPC-related NACs as may be expected but rather clusters with the earlier and later Hamburgian (Havelte) of the same microregion. This again may point to expert-biases but, given the large time gap between them, also towards processes of convergent evolution arising from comparable economic strategies focused on high mobility and reindeer hunting.

The *Technology* dendrogram further illustrates this complexity, revealing some interesting regional groupings (Fig 5B). The lower supercluster is mainly made up of NACs with north-eastern and central European provenience (Lithuania, Poland and Bohemia and Moravia), either indicating the structuring role of regional research traditions not as readily identifiable in the *Tools* data or pointing to an important superregional context of shared technological knowledge and practice. Noticeable is also the placement of most FBT/LBI-related NACs outside of the upper, better-defined supercluster. The two deviations to this tendency are probably significant as they pertain to the Epi-Ahrensburgian from Belgium/the Southern Netherlands attributed to TS III and IV. In contrast, while the position of the Epi-Ahrensburgian from Poland cannot be resolved, the Polish Swiderian and Pludian cluster closely together with northern FBT/LBI-associated complexes such as the Laborian, Belloisian and the British Long Blade Industries/Epi-Ahrensburgian, in line with previous arguments as to the technological similarities arising at this time [27,71,74]. Another important pattern in the *Technology* dataset is the placement of NACs attributed to the Mesolithic macro-unit in the upper supercluster. The only exception to this pattern is the Mesolithic from Bohemia and Moravia which instead clusters in a regionally circumscribed cluster. This possibly results from the mixing of Epimagdalenian (layer 4) and Mesolithic (layer 3) layers of the Kůlna Cave—the key site representing the earliest Mesolithic in the area, thus imitating technological continuity. Further technological studies on the Mesolithic of this region are required to solve this issue.

The Epigravettian is split into two groups, separating the Epigravettian-related NACs from north-eastern Italy and the region comprising Austria, Slovenia and Hungary. This, together with observations made above, suggests that the Epigravettian requires robust re-evaluation. Overall, similarity in the *Technology* dataset is structured differently than in the *Tools* data and this may suggest that technologies and toolkit compositions are subjected to different evolutionary processes, with technological parameters often shared across large spatial scales while toolkits structure the record on smaller scales, at least in the latter half of the study period.

The *Outlines* dendrogram (Fig 5C), finally, appears at first sight to be poorly structured vis-à-vis assigned macro-units. This is likely an artefact of the stratified subsample used to construct it (see Methods). The dendrogram does reproduce some of the NAC relationships already outlined. For example, Epigravettian and the Mesolithic armature outlines largely spread across the entire dendrogram, although the latter also displays some clustering, especially its southern and central European taxa. There is notable clustering of TPC in the upper part of the dendrogram and this occurs in the neighbourhood of shouldered point-related NAC instances, for example of the Hamburgian; tanged and shouldered points cannot always be clearly distinguished on shape alone. As in the *Tools* and *Technology* datasets, the position of the Epi-Ahrensburgian—especially in the Low Countries—poses important questions but, in contrast to the other data domains, it is not obvious whether Epi-Ahrensburgian armature shapes are closer related to the TPC or other FBT/LBI-related armatures. The status of Epi-Ahrensburgian is ambiguous and this may in part echo the already ambivalent qualification as ‘epi-’ as applied to some of the concerned assemblages, which is often taken to signal both continuity/similarity and discontinuity/dissimilarity at the same time.

As before, consistently clustered NACs are often geographically and chronologically close but were in most instances submitted by the same experts. They also tend to include a range of taxa that are not semantically or chronologically close. It is therefore generally difficult to disentangle the effects of culture-historical relatedness generated by shared lithic traditions and close contacts between the respective hunter-gatherer populations from the combined effects of convergent developments of similar armature shapes and a systematic bias introduced by research history and a priori assumptions about lithic technology in those areas.

**Fig 6:**
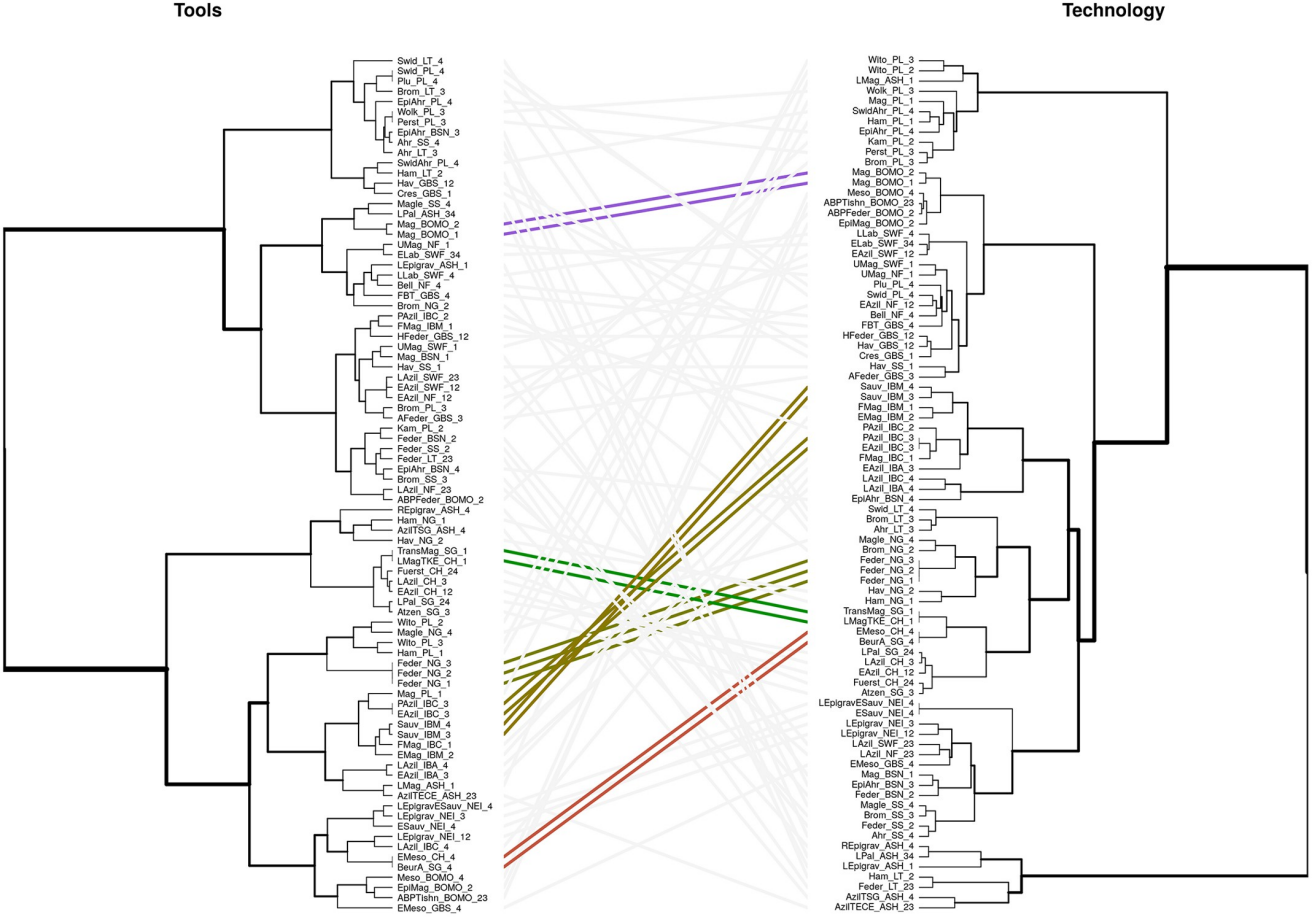
Tanglegram of Tools (left) and Technology (right) non-bootstrapped dendrograms, constructed using the cophyloplot function in ape [149]. The topography of the two dendrograms differs and only few cultural taxonomic units occur in the same clades. Those NACs which occur in the same clade are coloured.

Fig 6 compares the *Tools* and *Technology* datasets directly by constructing a so-called tanglegram—a juxtaposition of two structures contained in separate dendrograms that allows the matching of topological features between different domains [for earlier applications in archaeology see 165,166]. This *Tools-Technology* tanglegram reveals that only few NACs consistently occur together, albeit at different places within the tree topologies. This underlines that the two domains of lithic variability—toolkit composition and laminar reduction technology—are not structured in a similar way and that they may be subjected to different processes and modes of evolution. Our data are not consistent with a tightly interlinked co-evolution between the two domains and instead point to disjunct dynamics of long-term change, possibly with changing relationships between prevalent tool morphotypes and techno-economic principles of laminar reduction.

**Fig 7:**
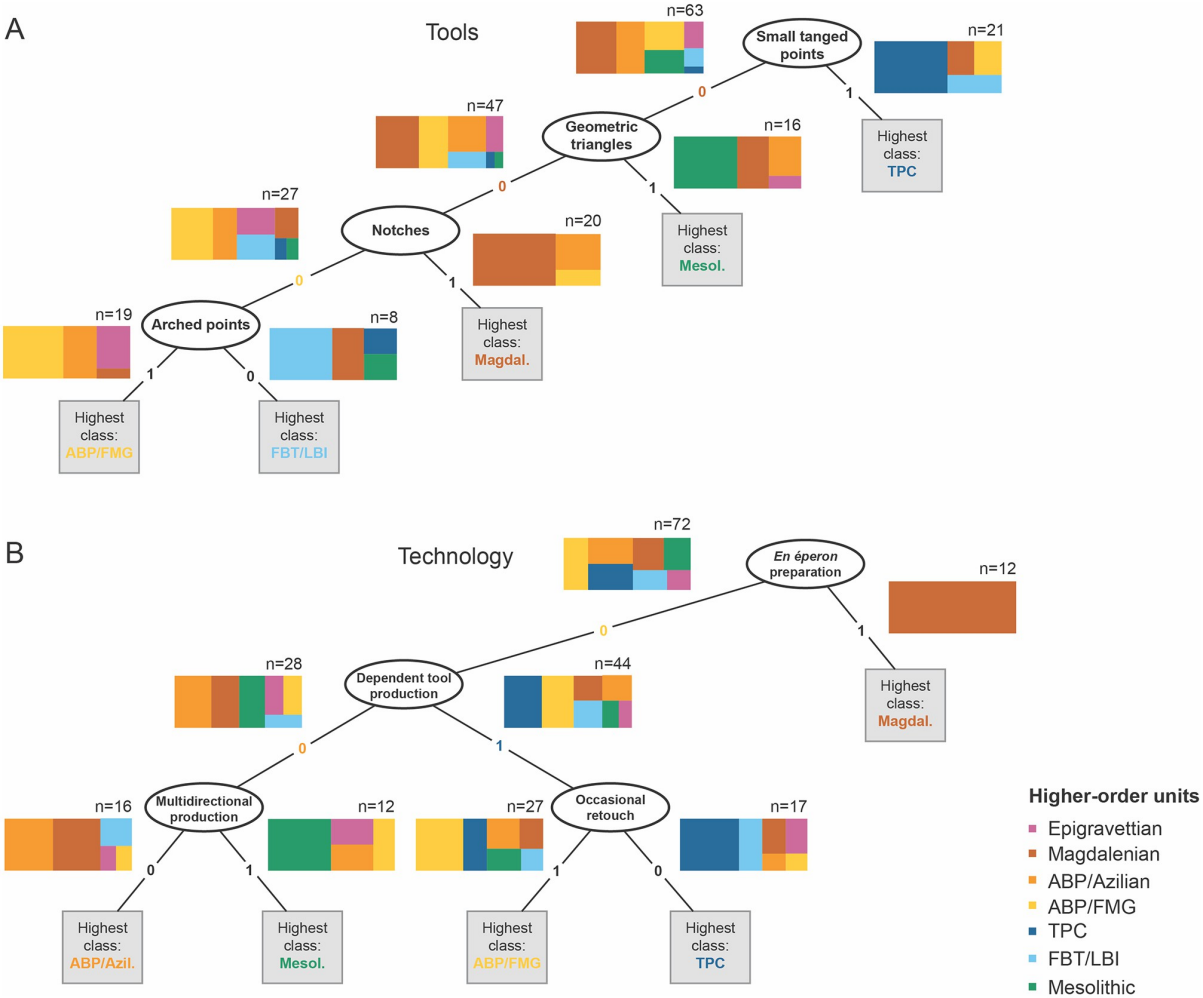
Machine-learning classification and regression trees (CART) for Tools (A) and Technology (B) vis-à-vis higher-order taxonomic groupings. The two decision trees show the most important predictor variables and their character states in our dataset for the higher-order archaeological groupings in each lithic data-domain. Each step in the tree splits the remaining units from the previous subset according to the identified key variable. Colour-coded treemaps show the precision and importance of the any given variable’s character state for classifying the seven higher-order taxonomic groupings in the dataset. CART was performed with ‘Partial data’ and ‘Minimum error’ pruning in R and then redrawn from DisplayR.

Fig 7 shows the results of the machine learning classification and regression trees for the *Tools* and *Technology* datasets (detailed results and observations provided in Supplementary Information S5.1 in S5 Data). They confirm that both datasets discriminate differently between the higher-order macro-units. While the *Tools* data produces a strictly unilineal decision tree, the *Technology* data yields a bifurcated tree with two separate pairs of macro-units defined rather differently: blank-tool dependencies broadly characterize ABP/FMG and TPC, which in turn can often be distinguished by differential assemblage-wide retouch intensities, whereas ABP/Azilian and the Mesolithic tend to be based on a technological disjunction between blank and tool production but can typically be disaggregated by the importance of multidirectional core exploitation. More generally, the results show that both data domains are differentially suitable to recover the target macro-units and different units are more readily recovered by either *Tools* or *Technology* data. Different *Tools* and *Technology* traits describe the different macro-units and the most relevant are portrayed in Fig 7. The Magdalenian is, for example, set apart by a strong proclivity towards *en éperon* platform preparation. Some units are mostly defined by the *absence* of particular traits, such as FBT/LBI with regard to *Tools* and ABP/Azilian with regard to *Technology*. This negative definition of FBT/LBI in terms of *Tools* traits may at first glance be surprising given the previously reported elevated toolkit discriminability of this macro-unit. The CART results, however, merely suggest that there is no single toolkit variable or a layering of specific variables whose presence or absence reasonably predict an FBT/LBI attribution. Together with the other results reported for the FBT/LBI in this study, this suggests that the macro-unit is well-defined by its *difference* from other well-defined macro-units but that this discriminability is not linked to a positive definition of toolkits in terms of the presence of certain derived tool forms. This finding clearly supports the priority currently given to a broadly technological definition of this macro-unit. Again, the Epigravettian macro-unit is difficult to recover from both data domains, but this is probably the result of the small sample size for this unit and its evident longevity across all time-slices considered here.

**Fig 8:**
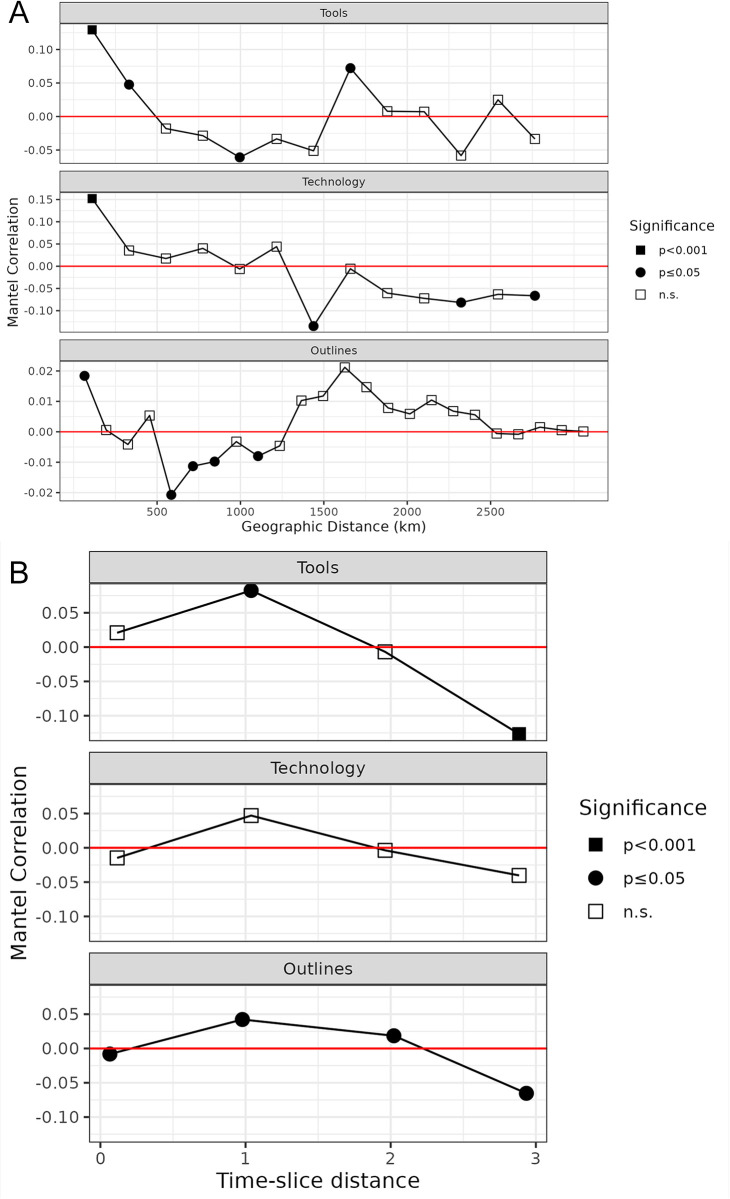
**A.** Mantel tests for the three analytical domains testing the null hypothesis that between-data similarity (NAC-level for Tools and Technology and site-level for Outlines) decreases with geographical distance. **B.** Mantel tests for the three analytical domains testing the null hypothesis that between-data similarity (NAC-level for Tools and Technology and site-level for Outlines) decreases with time-slice distance.

Results obtained from Mantel correlograms are shown in Fig 8A and 8B. The results are consistently significant for the closest geographical NAC-neighbours across all three data domains (Fig 8A), thus supporting the notion that geographic distance is a generative factor in material culture similarity [cf. 167,168]. By the same token, however, the Mantel test results also show a complex pattern of non-linearity when it comes to the link between lithic similarity and geographic distance, and the patterns are not strongly congruent between the three data domains. It is interesting to note that significance is sometimes reached at geographic distances well beyond 500 km, with the *Tools* and *Technology* data, in contrast to the *Outline* data, even revealing significant relationships beyond 2000 km. The *Technology* data produces significant results at the largest distances. Although this signal is weak, it may indicate that traits and knowledge pertaining to toolkits and especially production technologies have larger geographic reach than tool shapes which may be more responsive to functional requirements tied to region-specific ecologies and raw material constraints—although this may also invite very particular interpretations of cultural contact across these distances given that the details of flint knapping may not be the most readily visible and hence unlikely to be shared through casual contact only [cf. 169]. To assess to what degree temporal rather than spatial distance contributes to the structuring of the lithic data at hand, we conducted Mantel tests on the relationship between similarity and time-slice distance, and these yield significant relationships especially for *Outlines* data and to some extent for *Tools* (Fig 8B), reinforcing the notion that that there is some time-dependency in the evolution of lithic tool shape design. The *Technology* data, by contrast, do not yield such a pattern and this may suggest that similarity in this data domain is mainly structured synchronically and super-regionally.

The Mantel correlograms are ultimately difficult to interpret because of the many confounding factors including human demography likely playing into these patterns, and because of the possibility of convergent evolution in lithic technology and morphology [170,171, e.g. 172]. This being said, the alternation between significant and non-significant distance-dependent geographic similarity in the data most likely relates to the flow of materials, artefacts and information with changing network structures [e.g. 173–176]. However, distance-similarity relationships are not stable across time and future work could attempt to tease these factors apart to further qualify our findings. It is interesting to note here that the structure revealed by the *Technology* dendrogram reported above entails a broad supra-regional dimension: the lower clade seems to circumscribe a larger area that can broadly be equated with the Northern and North-eastern European Plain including Southern Scandinavia, Northern Germany, the Baltic region and Poland, while the upper clade comprises macro-regions further to the south and in western Europe (cf. Fig 6). This distinction broadly corresponds with the geographic extent of TPC and FBT/LBI-related NACs in north-western, northern, and eastern Europe.

**Fig 9:**
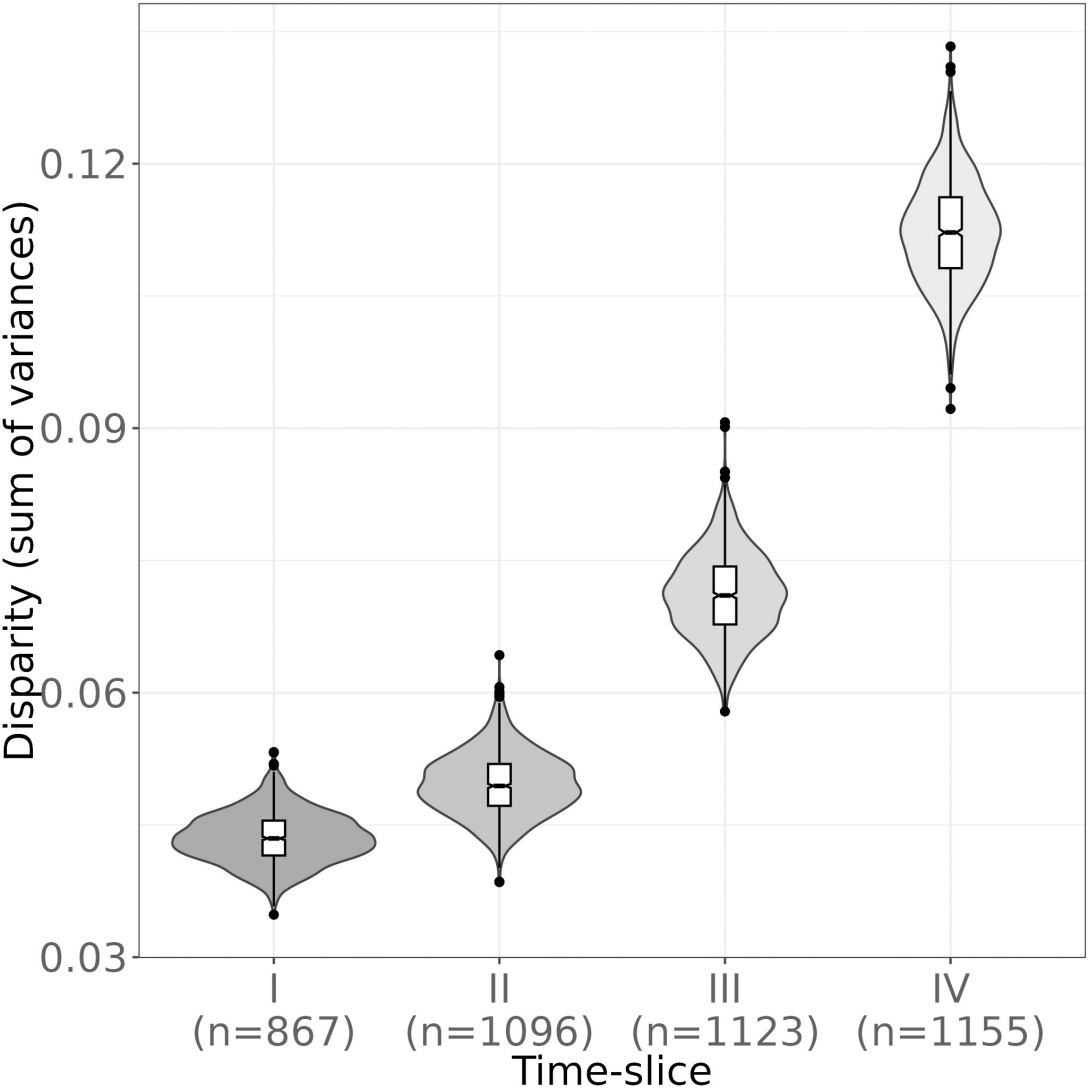
Disparity of lithic armature outlines across time (time-slices I to IV).

Finally, Fig 9 presents the chronological development of measured disparity in lithic armatures based on their outlines [e.g. 173–176]. There is a clear trend: the sum of 2D-shape variance remains stable across TS I and II but increases markedly in TS III and then again in TS IV. These differences are statistically significant (Supplementary Information S5.2 in S5 Data). Such emergent diversification of tool forms has long been recognized in the Final Palaeolithic and earliest Mesolithic [106,177,178]. This includes two aspects of hypothesised evolutionary change: 1) the pronounced lithic normativity that characterized much of the Middle and Upper Magdalenian is thought to be softened both in relation to tool forms and technological schemata [45,52]; and 2) a larger number of cultural taxa is said to arise towards the end of the Terminal Pleistocene and the early Holocene as the spatially expansive communities of practice commonly referred to as the Magdalenian and Epigravettian fragmented into less expansive regional units [106]. Our data is consistent with this scenario and suggests that this process developed considerably momentum with the emergence of TPC, FBT/LBI and Mesolithic-related NACs from TS III onwards. This increase in tool shape diversity may in turn be interpreted as a response to population growth towards the end of the Pleistocene followed by a demographic disruption and reorganisation in the context of the Younger Dryas.

## Discussion

Our aim has been to quantitatively assess the validity of current cultural taxonomic schemes for the Final Palaeolithic and earliest Mesolithic (15–11 ka cal BP) in Europe and on this basis to infer patterns and processes of material culture diversification, cultural transmission, and adaptation. To do so, we have collaboratively assembled a large and unique dataset encompassing information on lithic tools, knapping technology, and armature shapes, harnessing the first-hand expertise of regional specialists. Overall, the results of our macro-archaeological analyses are complex, surprising, and to some extent sobering. Deploying and extending an analytical approach that has been validated against previously published data from other periods and regions [141], our results confirm the broad heuristic utility of some traditional named cultural taxonomic groupings (e.g. the Magdalenian, the Ahrensburgian), while failing to robustly replicate others (e.g. the proposed taxonomic distinctions in the Lithuanian inventories, the Brommean). Our results provide both a hopeful and a cautionary tale, and while it may be tempting to interpret this outcome as support for critiques of the cultural taxonomy of much of the Palaeolithic as so many ‘accidents of history’ [2], a swathe of other factors must also be considered. Firstly, the degree to which different material culture domains are amenable to coherent taxonomic assessments across data domains may inherently be limited. For instance, Roux and colleagues [179,180], Kuhn [181], and Perlès [182,183] have shown using a range of approaches that different aspects of material culture often do not change in lock-step, even when assigned to the same overarching cultural groups or taxa. Specifically for the Final Palaeolithic of Europe, similar arguments have been put forward for understanding the interrelationship between osseous projectiles and lithic technology [184,185], and between artistic practices and lithic technology [186]. In addition, Grøn et al. [187,188] have shown on the basis of ethnoarchaeological data that individuals at times deliberately diverge from supposed material culture schemata, and this idea has subsequently been transferred to the Final Palaeolithic in an attempt to explain the substantial artefact shape and technological variation observed in the arch-backed point complex [58]. In this manner, our analyses do not support the notion that material culture and especially lithic projectile points acting as unequivocal social or territorial markers across this time.

The hierarchical patterns—and their absence—emerging in each of our three datasets are most likely, in our view, the result of the different generative processes that acted on material culture variability in the past on the one hand, and differences in classification practices across regions and research schools on the other. There exist, for example, marked differences in classificatory practice between Anglophone and Francophone traditions in European lithic research [189], although these are perhaps not as pronounced in regard to the Final Palaeolithic and earliest Mesolithic as in other periods. Other classificatory practices such as the one founded by Laplace [107] in Italy [see 190] or the Eastern European approach that variously emphasised quantitative and qualitative-technological analyses [e.g. 191], introduce additional variability.

In our analysis, taxa submitted by the same expert often cluster together independently of semantic distance; at the same time, geographically close taxa also often cluster together—those from the British Isles often are found close to those from Northern France, those from Northern Germany often close to those from Southern Scandinavia—and this likely is the combined result of shared classificatory practices as well as actual cultural affiliations of the ancient flintknappers whose products are under study here. Furthermore, some hypothesized higher-order groupings perform surprisingly well and appear to be a useful heuristic tool to discuss techno-cultural variability in the European Final Palaeolithic and earliest Mesolithic. Still, idiosyncratic differences in character state coding for the presence and absence of lithic retouched tools and for technological attributes may act as strong confounders. Furthermore, the analytical detail available even to experts varies across regions due to preservation and publication biases and because time-consuming aspects such as technological analyses have been in greater focus in some areas rather than others [see 192]. Finally, there are also important differences in site qualities across regions.

Finally, the clear trends towards greater armature diversity over time asks for further interrogation. As shown by Matzig and colleagues [141], disparity values may be interpreted as the morphometric equivalent of the coefficient of variation, which in turn may provide insights into social transmission pathways [161,162,193]. Low disparity values may be indicative of highly normative and largely vertical social transmission, as may be the case in the Magdalenian. High disparity scores are more difficult to interpret on their own. If internal ordering can be recognised, high disparity values would suggest cultural diversification, whereas high disparity values in the absence of internal structuring to this variation would instead suggest low-fidelity or low-normativity social transmission. In our view, the rising disparity scores during TS II and III indeed indicate social transmission modalities significantly less formal compared to the Magdalenian, whereas the elevated disparity score evident in TS IV may additionally hint at the more concerted regional differentiation of cultural expressions in the early Holocene.

## Conclusion

The assembly of a unique, expert-sourced and near-continental scale dataset on typological and technological developments in the Final Palaeolithic and earliest Mesolithic of Europe (c. 15–11 ka cal BP) has allowed us to quantitatively assess the degree to which traditional cultural taxonomic classifications may be replicated in a data-driven manner. Our study was designed with synthesis in mind and as an attempt to overcome the known conceptual and analytical heterogeneity that characterises lithic analysis. Overall, such a large-scale replication proved complicated and was only partially successful. In working through the issue of Final Palaeolithic/earliest Mesolithic cultural taxonomy, differences in terminology and epistemology became clear, even just within the author group of the present paper. By this token, a plethora of confounding factors—differences in research traditions, geography, and inter-observer variability—have likely introduced biases into the dataset that remain difficult to tease apart. It is not evident from our analysis whether an accounting of retouched tool classes, technological traits or lithic armature shapes would provide more robust entries into Final Palaeolithic/earliest Mesolithic cultural taxonomy. We thus caution against the use of such constructs as units of analysis rather than treating them as the abstractions they are. Different units are differentially sensitive to different lithic data domains and individual traits. Our analysis does, however, support a diversification of material culture in the realm of armature shapes towards the end of the Palaeolithic. This may relate to the marked changes in environmental pressures and their spatial extent at the transition from the Pleistocene into the Holocene [cf. 194], which likely precipitated changes in socio-ecological organisation, interaction, and hence technology. We also capture non-linear relationships between different domains of analysis such as toolkit composition, laminar production technology, and individual artefact forms. Future research should address this compositional complexity. In sum, our work aligns with approaches to lithic tool shape and technological analyses that—especially if integrated with quantitative and replicable protocols—promise an understanding of material culture change beyond typological categories and traditionally named units [195–199].

In closing, it is valuable to recall that the Final Palaeolithic and earliest Mesolithic are not the only periods of the deep past for which issues of cultural taxonomy are critically discussed. Similar uncertainties beleaguer the Levantine Mousterian [200,201] as well as the African Stone Age where named ‘complexes’ or ‘cultures’ such as the Nubian [170] and other NACs [202,203] are strongly contested. Other examples are the various divisions put forward for the Early Upper Palaeolithic Aurignacian or between the Levantine Aurignacian and the so-called (Early) Ahmarian [cf. 204,205] as well as the many intensely debated ‘transitional’ or Initial Upper Palaeolithic industries [102,206,207]. Likewise, the mid-Upper Palaeolithic Gravettian is divided by some into a plethora of different units variously defined on the presence or absence of particular lithic elements [e.g. 208–210]. Cultural taxonomic debates also occur on the other side of the Pleistocene-Holocene divide where the sheer number of NACs rises steeply while their epistemological status often remains far from clear [e.g. 211]. All of this seems to suggest that it remains as important to work on the pertinent theoretical, conceptual and interpretive aspects of such designations [cf. 212,213] as it is to experiment with novel quantitative approaches that allow integration of large datasets and facilitate data interoperability and replicability.

The study presented here does not provide an analytical panacea to any of these cultural taxonomic quandaries. However, it does highlight the complexity and differential utility of current taxonomic practices and thus stresses the urgent need to discuss and develop approaches to lithic data generation and analysis that facilitate robust and replicable syntheses. Improvements in study design and data curation across research traditions and infrastructures would strongly facilitate investigations of long-term and superregional constellations of technology, cultural transmission, and ecological adaptation as well as the role and scope of regionally specific culture histories. Vitally, ethnographically informed work [214,215] and emerging paleogenetic data increasingly call into question the existence of distinct, regionally-specific forager subpopulations, both in general [216] and specifically with regard to the European Terminal Pleistocene [217]. In this context, it is interesting to note the recently suggested connections between the Epigravettian and Azilian/Federmessergruppen as traditionally defined and the consistent clustering of artefacts associated with these cultural taxonomic groups in our analysis. This may be taken as tentative support for biocultural connections among these populations. Going forward, it becomes a critical matter to more precisely define how if at all traditional named archaeological cultures provide meaningful analytical units for the genetically, culturally, and ecologically fluid worlds of Pleistocene mobile foragers. Improved data-driven cultural taxonomic precision may allow us to better align and thereby mutually enrich the many emerging datasets—climatic, genetic, and archaeological—that allow us to address these dynamics.

## Supporting information

S1 Data Regional units of analysis and key sites. (DOCX)

S2 Data Site quality. (DOCX)

S3 Data Data description. (DOCX)

S4 Data Artefact outline data. (DOCX)

S5 Data Additional results. (DOCX)
