# Correction: A quantitative analysis of Final Palaeolithic/earliest Mesolithic cultural taxonomy and evolution in Europe

**DOI:** 10.1371/journal.pone.0315964

**Published:** 2024-12-12

**Authors:** Felix Riede, David N. Matzig, Miguel Biard, Philippe Crombé, Javier Fernández-Lopéz de Pablo, Federica Fontana, Daniel Groß, Thomas Hess, Mathieu Langlais, Ludovic Mevel, William Mills, Martin Moník, Nicolas Naudinot, Caroline Posch, Tomas Rimkus, Damian Stefański, Hans Vandendriessche, Shumon T. Hussain

There are errors in the Author Contributions. The correct contributions are:

**Conceptualization:** Felix Riede, David N. Matzig, Shumon T. Hussain.

**Data curation:** David N. Matzig, Shumon T. Hussain.

**Formal analysis:** David N. Matzig, Shumon T. Hussain.

**Funding acquisition:** Felix Riede.

**Investigation:** Felix Riede, Miguel Biard, Philippe Crombé, Javier Fernández-Lopéz de Pablo, Federica Fontana, Daniel Groß, Thomas Hess, Mathieu Langlais, Ludovic Mevel, William Mills, Martin Moník, Nicolas Naudinot, Caroline Posch, Tomas Rimkus, Damian Stefański, Hans Vandendriessche, Shumon T. Hussain.

**Methodology:** Felix Riede, David N. Matzig, Shumon T. Hussain.

**Project administration:** Felix Riede, David N. Matzig, Shumon T. Hussain.

**Resources:** Felix Riede.

**Software:** David N. Matzig, Shumon T. Hussain.

**Supervision:** Felix Riede, Shumon T. Hussain.

**Validation:** Felix Riede, Miguel Biard, Philippe Crombé, Javier Fernández-Lopéz de Pablo, Federica Fontana, Daniel Groß, Thomas Hess, Mathieu Langlais, Ludovic Mevel, William Mills, Martin Moník, Nicolas Naudinot, Caroline Posch, Tomas Rimkus, Damian Stefański, Hans Vandendriessche, Shumon T. Hussain.

**Visualization:** Felix Riede, David N. Matzig, Shumon T. Hussain.

**Writing–original draft:** Felix Riede, David N. Matzig, Shumon T. Hussain.

**Writing–review & editing:** Felix Riede, David N. Matzig, Miguel Biard, Philippe Crombé, Javier Fernández-Lopéz de Pablo, Federica Fontana, Daniel Groß, Thomas Hess, Mathieu Langlais, Ludovic Mevel, William Mills, Martin Moník, Nicolas Naudinot, Caroline Posch, Tomas Rimkus, Damian Stefański, Hans Vandendriessche, Shumon T. Hussain.
