# Supplementary material for: A quantitative analysis of Final Palaeolithic/earliest Mesolithic cultural taxonomy and evolution in Europe
Source: PLoS One. 2024 Mar 11;19(3):e0299512. doi: 10.1371/journal.pone.0299512 (PMC10927100; doi:10.1371/journal.pone.0299512)
Supplement: S1 Data — (DOCX) [file pone.0299512.s001.docx]

**ELECTRONIC SUPPLEMENTARY MATERIALS of**

*Riede et al., A quantitative analysis of Final Palaeolithic/earliest Mesolithic cultural taxonomy and evolution in Europe*

************************************************************

Supplementary Information S1: Regional units of analysis and key sites

**S1 Table 1**. Overview of the regional units of analysis, their corresponding region code and expert ID.

| **#** | **Region** | **Region code** | **Expert ID** |
| --- | --- | --- | --- |
| 1 | Southern Scandinavia | SS | FR |
| 2 | Lithuania | LT | TR |
| 3 | Northern Germany | NG | DG |
| 4 | Britain | GBS | MW |
| 5 | Poland | PL | SD |
| 6 | Belgium and Southern Netherlands | BSN | HV, PC |
| 7 | Bohemia and Moravia | BOMO | MM |
| 8 | Southern Germany | SG | TH |
| 9 | Northern France | NF | LM, MB |
| 10 | Austria, Slovakia and Hungary | ASH | CP |
| 11 | Switzerland | SW or CH | TH |
| 12 | Northern/North-eastern Italy | NEI | FF |
| 13 | (South)Western France | SWF | ML, NN |
| 14 | Cantabrian Spain | IBC | JFLdP |
| 15 | Mediterranean Iberia | IBM | JFLdP |
| 16 | Atlantic Iberia | IBA | JFLdP |

**S1 Table 2**. The number of recorded key sites per macro-region used in this analysis. A full list of key sites can be extracted from the published dataset freely made available at <https://doi.org/10.5281/zenodo.7940337>). In Poland, single sites are resolved by layer/context such that the actual number of sites is lower.

| **Regional Unit** | **N_key sites_** |
| --- | --- |
| Southern Scandinavia | 33 |
| Lithuania | 17 |
| Northern Germany | 19 |
| Britain | 40 |
| Poland | 83 |
| Belgium and Southern Netherlands | 16 |
| Bohemia and Moravia | 12 |
| Southern Germany | 10 |
| Northern France | 9 |
| Austria, Slovakia and Hungary | 13 |
| Switzerland | 11 |
| Northern/North-eastern Italy | 13 |
| (South)Western France | 18 |
| Cantabrian Spain | 19 |
| Mediterranean Iberia | 30 |
| Atlantic Iberia | 7 |
| *total* | **350** |


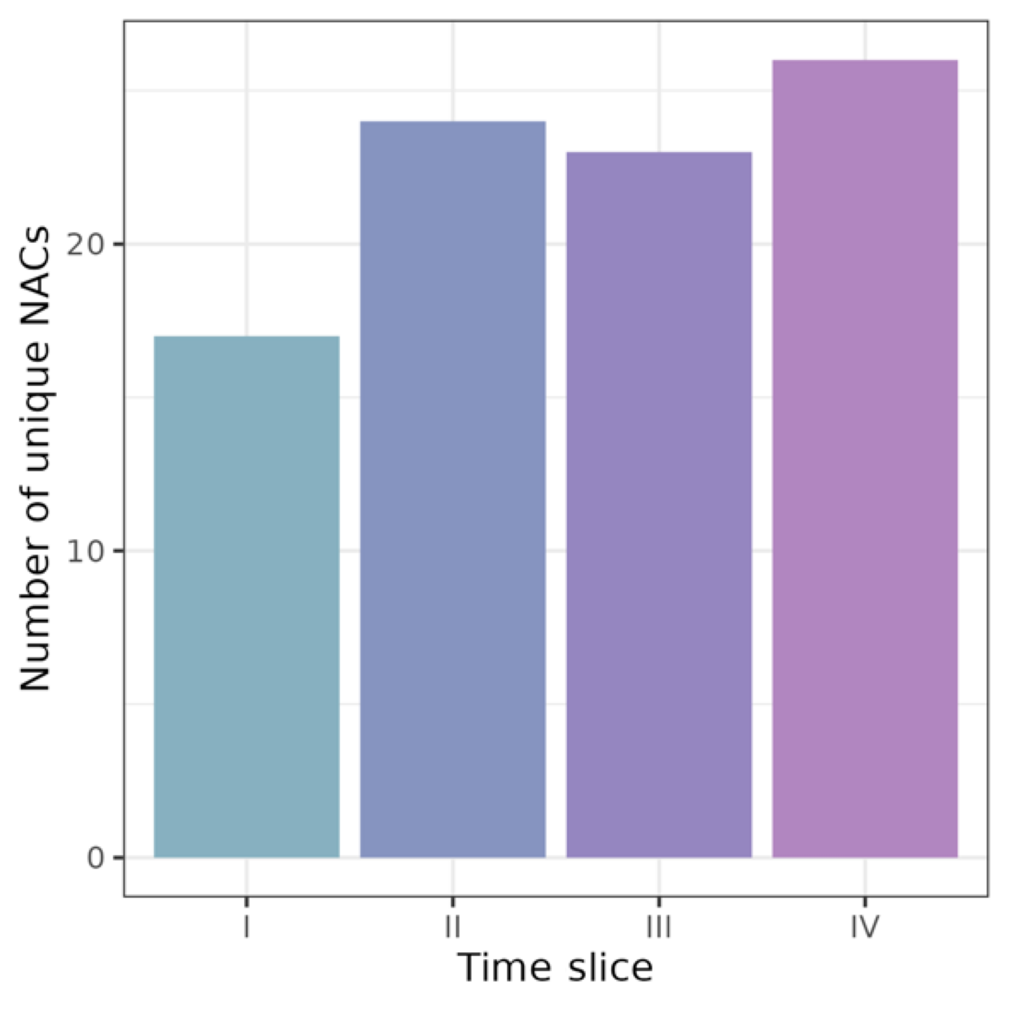


**S1 Figure 1.** Frequency of unique NACs from time slice I to IV. NAC scores are based on the raw labels/names of taxonomic units as originally submitted by regional experts and contained in the non-translated database entries.
