# Supplementary material for: A quantitative analysis of Final Palaeolithic/earliest Mesolithic cultural taxonomy and evolution in Europe
Source: PLoS One. 2024 Mar 11;19(3):e0299512. doi: 10.1371/journal.pone.0299512 (PMC10927100; doi:10.1371/journal.pone.0299512)
Supplement: S2 Data — (DOCX) [file pone.0299512.s002.docx]

**ELECTRONIC SUPPLEMENTARY MATERIALS of**

*Riede et al., A quantitative analysis of Final Palaeolithic/earliest Mesolithic cultural taxonomy and evolution in Europe*

************************************************************

Supplementary Information S2: Site quality

Data quality is assessed on the level of individual archaeological sites and presented as a “Quality_Score”. The scoring procedure is based on site-specific metadata, e.g. the nature of the archaeological deposits/layers in question, estimated assemblage coherence, and the year of excavation (for details, see Supplementary information S3.4).


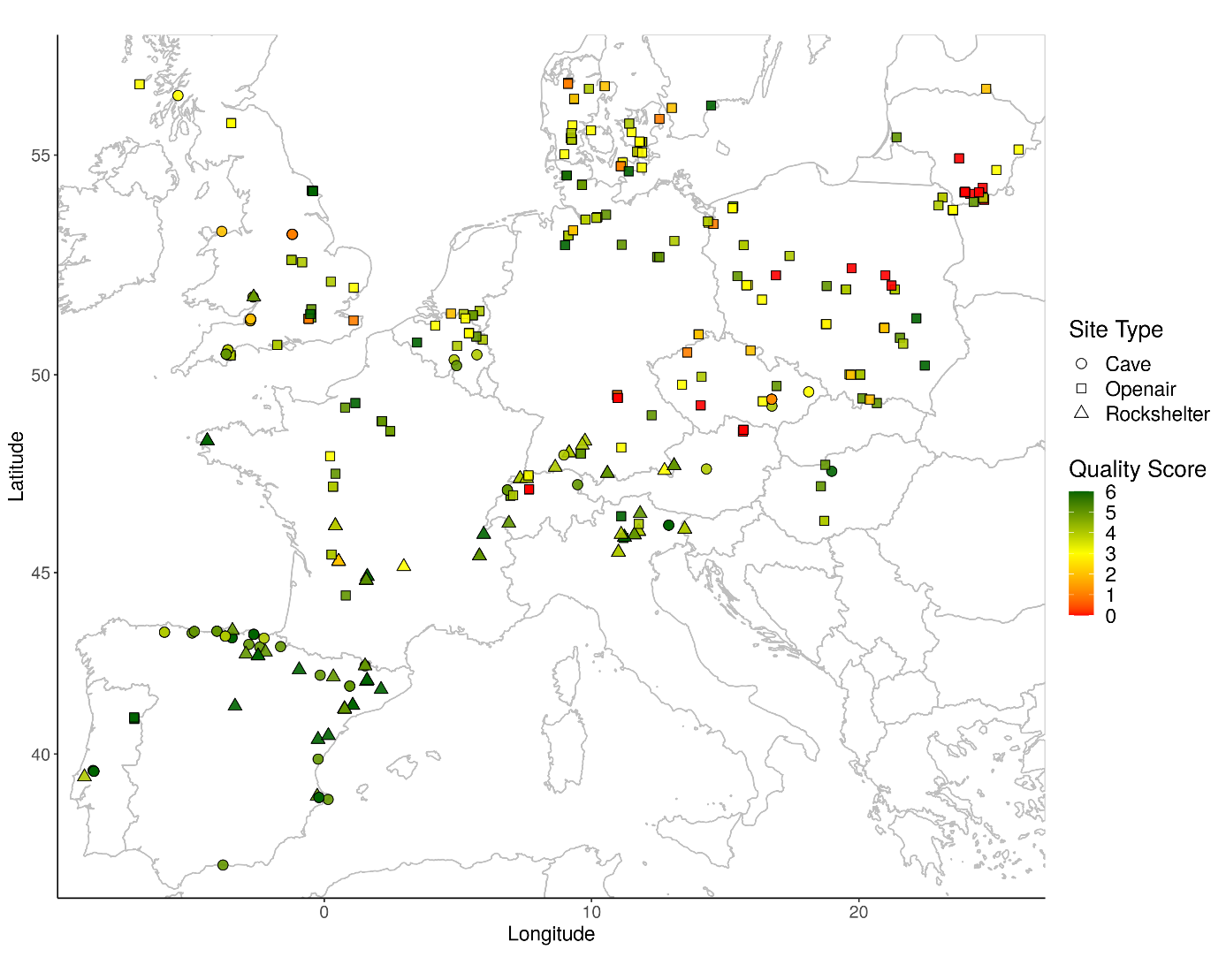


**S2 Figure 1a**. All key sites included in the database and their associated quality scores.


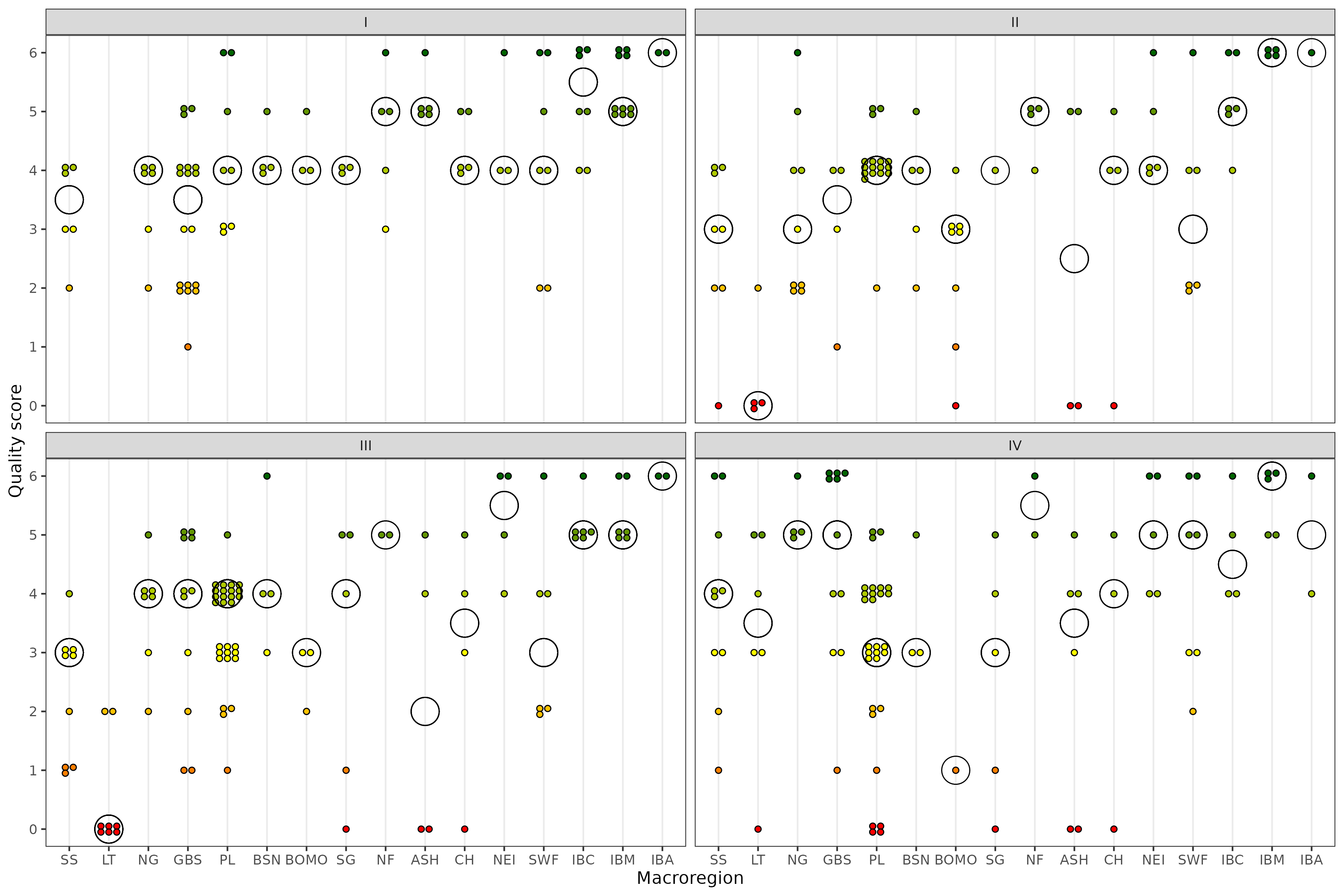


**S2 Figure 1b**. Median quality-scores per region and time-slice.

**S2 Table 1:** Median quality score per region per time-slice.

| Region code (N_sites_) | Time-slice | Median quality score |
| --- | --- | --- |
| ASH (n=5) | I | 5 |
| BSN (n=4) | I | 4 |
| BOMO (n=3) | I | 4 |
| NEI (n=3) | I | 4 |
| NG (n=6) | I | 4 |
| SG (n=3) | I | 4 |
| CH (n=5) | I | 4 |
| SWF (n=7) | I | 4 |
| PL (n=8) | I | 4 |
| IBC (n=6) | I | 5.5 |
| IBM (n=10) | I | 5 |
| IBA (n=2) | I | 6 |
| SS (n=6) | I | 3.5 |
| NF (n=5) | I | 5 |
| GBS (n=18) | I | 3.5 |
| ASH (n=4) | II | 2.5 |
| BSN (n=5) | II | 4 |
| BOMO (n=8) | II | 3 |
| LT (n=4) | II | 0 |
| NEI (n=5) | II | 4 |
| NG (n=9) | II | 3 |
| CH (n=4) | II | 4 |
| SG (n=1) | II | 4 |
| SWF (n=6) | II | 3 |
| PL (n=17) | II | 4 |
| IBC (n=6) | II | 5 |
| IBM (n=4) | II | 6 |
| IBA (n=1) | II | 6 |
| SS (n=8) | II | 3 |
| NF (n=4) | II | 5 |
| GBS (n=4) | II | 3.5 |
| SS (n=9) | III | 3 |
| ASH (n=4) | III | 2 |
| BSN (n=4) | III | 4 |
| BOMO (n=3) | III | 3 |
| LT (n=8) | III | 0 |
| NEI (n=4) | III | 5.5 |
| NG (n=7) | III | 4 |
| CH (n=4) | III | 3.5 |
| SG (n=5) | III | 4 |
| SWF (n=6) | III | 3 |
| PL (n=29) | III | 4 |
| IBC (n=6) | III | 5 |
| IBM (n=6) | III | 5 |
| IBA (n=2) | III | 6 |
| NF (n=2) | III | 5 |
| GBS (n=11) | III | 4 |
| ASH (n=6) | IV | 3.5 |
| BSN (n=3) | IV | 3 |
| BOMO (n=1) | IV | 1 |
| LT (n=6) | IV | 3.5 |
| NEI (n=5) | IV | 5 |
| NG (n=4) | IV | 5 |
| CH(n=3) | IV | 4 |
| SG (n=5) | IV | 3 |
| SWF (n=7) | IV | 5 |
| PL (n=29) | IV | 3 |
| IBC (n=4) | IV | 4.5 |
| IBM (n=5) | IV | 6 |
| IBA (n=2) | IV | 5 |
| SS (n=10) | IV | 4 |
| NF (n=2) | IV | 5.5 |
| GBS (n=11) | IV | 5 |
