# Supplementary material for: A quantitative analysis of Final Palaeolithic/earliest Mesolithic cultural taxonomy and evolution in Europe
Source: PLoS One. 2024 Mar 11;19(3):e0299512. doi: 10.1371/journal.pone.0299512 (PMC10927100; doi:10.1371/journal.pone.0299512)
Supplement: S3 Data — (DOCX) [file pone.0299512.s003.docx]

**ELECTRONIC SUPPLEMENTARY MATERIALS of**

*Riede et al., A quantitative analysis of Final Palaeolithic/earliest Mesolithic cultural taxonomy and evolution in Europe*

************************************************************

Supplementary Information S3: The CLIOARCH 1511NAC Database

1. ***Database-wide information*** *to link relevant data entries*

Provided as numbers and as text entries.

| **Code** | **Description** |
| --- | --- |
| KeySite | Name of selected key site |
| Level/layer/ concentration | Context of selected lithic assemblage from key site |
| Site_ID | Unique site identifier |
| TaxUnit | Name of associated archaeological taxonomic unit |
| TaxUnit_unique | Unique TaxUnit identifier |
| Timeslice | Time-slice attribution (“1”, “2”, “3” or “4”); if a given TaxUnit spans multiple Timeslices, these a listed as comma separated numbers (e.g. “1,2”) |
| Expert_editor | Code for data provider |
| Macro_region | Macro-region for which lithic data was collected (frame of reference for TaxUnit determination) |
| Macro_region_code | Unique identifier of macro-regions |

1. ***Tool class data module***

Categorical information, recorded as binary data (“1” = recurrent/systematic presence; “0” = recurrent/systematic presence not attested; “N/A” = information not available).

Tool-class data operates on the aggregate level, recorded information refers to macro-categories of tools, not necessarily individual “types”.

*Armature/points*

| **Code** | **Description** |
| --- | --- |
| A_p | Simple points |
| A_ltangedp | Large tanged points (width >15mm) |
| A_stangedp | Small tanged points (width <15mm) |
| A_shoulderedp | Shouldered points |
| A_archedp | Arched/Arch-backed points |
| A_dejectp | Dejected/Angle-backed points |
| A_backed | (Other) Backed pieces |
| A_serrated | Serrated/denticulated implements |
| A_seg/lun | Segments/lunates |
| A_geom_rec | Rectangular (geometric) microliths |
| A_geom_trap | Trapezoid (geometric) microliths |
| A_geom_trian | Triangular (geometric) microliths |
| A_ventral | Partial ventral retouch |
| A_bifacial | Complete bifacial retouch (shaping)* |

** removed from the final dataset due to a lack of “1” entries*

*Domestic tools*

| **Code** | **Description** |
| --- | --- |
| D_endscrapers | Endscrapers |
| D_burins | Burins |
| D_borers | Borers (including becs and other atypical forms) |
| D_zinken | Zinken |
| D_notches | Notched pieces |
| D_denticulates | Denticulated pieces |
| D_combinatory/multi | Combinatory tools/multitools |
| D_knives | Large blade knives/heavily retouched larger blades |
| D_LCTs | (Other) Large Cutting Tools |
| D_adze/axe | Flake adzes/axes |

1. ***Technology data module***

Categorical information, recorded as binary data (“1” = recurrent/systematic presence; “0” = recurrent/systematic presence not attested; “N/A” = information not available).

Only few of these variables are (logically) mutually exclusive; in most cases, single variables are to be determined independently of all other variables.

Technology class data operates on the aggregate level, recorded information refers to technocomplex/taxon-wide trends and patterns, which are not necessarily reducible to each single site belonging to the unit in question.

*Laminar reduction strategies and core structure*

| **Code** | **Description** |
| --- | --- |
| LP_reduction_strat_1 | One single focal reduction strategy |
| LP_reduction_strat_2 | Two focal reduction strategies |
| LP_reduction_strat_3 | Three focal reduction strategies |
| LP_reduction_strat_4 | Four focal reduction strategies |
| LP_unid | Undirectional laminar production |
| LP_bid | Bidirectional laminar production |
| LP_multid | Multidirectional laminar production |
| LP_stable | Reduction patters are stable across core life-histories |
| LP_changing | Reduction patterns change across core life-histories |
| LP_core_flanks | Prepared/well-defined core-flanks |
| LP_core_foot | Prepared/defined core foot |
| LP_core_back | Prepared/defined core back |
| LP_core_wide | Wide reduction surfaces |
| LP_core_narrow | Narrow reduction surfaces |
| LP_core_parallel | Geometry of reduction surfaces highlights parallel configurations |
| LP_core_convergent | Geometry of reduction surfaces highlights convergent configurations |
| LP_core_irregular | Irregular reduction surface geometries |
| LP_surface | Surface exploitation of cores (only surface-near volumes are actively exploited) |
| LP_volume | Volume(tric) exploitation of cores (core reduction concepts in principle allow for the exploitation of whole core volumes) |
| LP_edge | (Narrow) Edge exploitation (production of blanks from natural or artificial narrow edges without exploitation of whole core volumes, including burin-like productions) |
| LP_striking_platform | Prepared striking platform of cores |
| LP_crested_blades | Crested/neocrested blades |
| LP_core_tablets | Core tablets |
| LP_overshoot_blades | Preparatory/corrective overshoot blades |
| LP_internal | Internal knapping (knapping gesture results in point of impact in the interior of the striking platform, often associated with deep blank platforms/butts and pronounced striking features such as bulbs) |
| LP_tangential | Edge-near/peripheral knapping (knapping gesture results in point of impact very close or at the core edge, often resulting in marginal blank platforms and diffuse striking features) |

*Raw material economy*

| **Code** | **Description** |
| --- | --- |
| RE_diverse_nospec | Focus on a diverse raw material base but no obvious specialization (i.e. no specific link between a particular raw material and a particular reduction strategy) |
| RE_diverse_spec | Focus on a diverse raw material base with evidence on specialization on (a) selected raw material(s) |
| RE_uniform_nospec | Focus on uniform raw material base yet no obvious specialization, that is adaptation of the knapping strategy to the given raw material (properties, forms) |
| RE_uniform_spec | Focus on a uniform raw material base with evidence for specialization, that is, adaptation of knapping strategies to raw material conditions/properties |
| RE_highqual_spec | Global focus on high quality raw material with evident adaptation of knapping strategies to their raw material properties |

*Blade structure and morpho-typology of laminar blanks*

| **Code** | **Description** |
| --- | --- |
| L_parallel | Parallel outlines |
| L_convergent | Convergent outlines |
| L_irregular | Irregular outlines |
| L_straight | Straight laminar profiles |
| L_curved | Curved laminar profiles |
| L_twisted | Twisted laminar profiles |
| L_plain | Plain platforms |
| L_dihedral | Dihedral platforms |
| L_facetted | Facetted platforms |
| L_eperon | *En eperon* platform preparation |

*Other/non-laminar blank production systems and interrelationships*

| **Code** | **Description** |
| --- | --- |
| FP_indep | Independent flake production |
| FP_embedded | Embedded flake production (dependent on laminar production(s)) |
| BP_indep | Independent bladelet production |
| BP_embedded | Embedded bladelet production (dependent on blade or flake production(s)) |
| TP_indep | Tool production/manufacture independent of blank production (i.e. no systematic link between specific blank and tool forms/types) |
| TP_dep | Tool production/manufacture dependent or “follows/ anticipates” blank production (i.e. evidence for a systematic link between specific blank and tool forms/types) |
| TP_seg | Segmentation of domestic tools and armature (blank-tool relations differ between the two, different blanks are selected and transformed in both cases, either within the same reduction strategy or the two tool categories are produced from separate reduction strategies) |

*Retouch tendencies + microburin*

(All artefacts recorded >2 cm)

| **Code** | **Description** |
| --- | --- |
| R_pervasive | Assemblage-level retouch is pervasive (>20%) |
| R_occasional | Assemblage-level retouch is occasional (10-20%) |
| R_rare | Assemblage-level retouch is rare (<10%) |
| Microburin | Microburin products and by-products |

1. ***Archaeological site/context data module***

In contrast to the previous two data modules, the site/context module compiles categorical and non-categorical information on the level of individual key sites chosen to represent larger taxonomic archaeological units. Contextual information is either given as numbers, text, a combination of the two, or as predefined categorical selections (drop-down).

*Basic site-specific information and data quality*

| **Code** | **Description** |
| --- | --- |
| Long | Longitude coordinates (given in decimal degree) |
| Lat | Latitude coordinates (given in decimal degree) |
| BP | Broad dating range in cal. BP, e.g. “15-13 calBP” |
| Dating_method | Dating method, e.g. “14C”, “TL”, “typology”, “technology”, “biostratigraphy” or “geology” |
| Dating_qual | Dating quality (drop-down): “Reliable” or “Problematic” |
| Site_type | Type of site (drop-down): “Openair”, “Cave” or “Rockshelter” |
| Site_strat | Stratification (or not) of site (drop-down): “Stratified” or “Surface” |
| Ass_pos | Assemblage position (drop-down): “Primary/insitu” or “Secondary/relocated” |
| Ass_coh | Assemblage coherence (drop-down): “Homogeneous” or “Mixed” |
| Site_fau | Presence/preservation of faunal material (drop-down): “Preserved” or “No” |
| Site_excav | Period of excavation (drop-down): “Before WW2”, “1950-1980”, “1980-2000” or “After 2000” |

*Calculated site-quality scores*

| **Code** | **Description** |
| --- | --- |
| Quality_Score | Total sum of following scores: “1” for Dating_qual = “Reliable”, “1” for Site_strat = “Stratified”, “1” for Ass_pos = “Primary/insitu”, “1” for Ass_coh = “Homogeneous” + “1” for Site_excav = “1980-2000” or “2” for Site_excav = “After 2000” (total possible score = “6”) |
| Quality_Rank | “1” if Quality_Score = 5-6, “2” if Quality_Score = 3-4, “3” if Quality_Score = 1-2 or “4” if Quality_Score = 0 |

*Register of processed and recorded tool outlines*

(Provided as “1” for available and “0” for not available)

| **Code** | **Description** |
| --- | --- |
| Outline_AR_TS1 | Armature outlines for Time-slice I |
| Outline_AR_TS2 | Armature outlines for Time-slice II |
| Outline_AR_TS3 | Armature outlines for Time-slice III |
| Outline_AR_TS4 | Armature outlines for Time-slice IV |
| Outline_ES_TS1 | Endscraper outlines for Time-slice I |
| Outline_ES_TS2 | Endscraper outlines for Time-slice II |
| Outline_ES_TS3 | Endscraper outlines for Time-slice III |
| Outline_ES_TS4 | Endscraper outlines for Time-slice IV |
| Outline_BR_TS1 | Borer outlines for Time-slice I |
| Outline_BR_TS2 | Borer outlines for Time-slice II |
| Outline_BR_TS3 | Borer outlines for Time-slice III |
| Outline_BR_TS4 | Borer outlines for Time-slice IV |

Note that in the present paper, only armature (AR) shapes are used.
