# Supplementary material for: A quantitative analysis of Final Palaeolithic/earliest Mesolithic cultural taxonomy and evolution in Europe
Source: PLoS One. 2024 Mar 11;19(3):e0299512. doi: 10.1371/journal.pone.0299512 (PMC10927100; doi:10.1371/journal.pone.0299512)
Supplement: S4 Data — (DOCX) [file pone.0299512.s004.docx]

**ELECTRONIC SUPPLEMENTARY MATERIALS of**

*Riede et al., A quantitative analysis of Final Palaeolithic/earliest Mesolithic cultural taxonomy and evolution in Europe*

************************************************************

Supplementary Information S4: CLIOARCH Outline database

1. ***Data structure and recording scheme***

The outline dataset consists of individual image files, which have been pre-processed into binary artefact images, which can directly be fed into an outline extraction and shape-description workflow (see detailed description of extraction and data preparation methods in the main paper). Original artefact imagery does not necessarily contain only a single lithic artefact but sometimes gather multiple objects as on many traditional artefact figures. The semi-automated extraction protocol generates individual files for each extracted object-outline (Matzig 2021a, b). It is these individual object-outline image files (images as data), which are stored in the database. Note that in the paper associated with this Supplementary Information, only the armature outlines are used.

The raw outline data can be found here: <https://doi.org/10.5281/zenodo.7940337>.

Individual outlines are grouped and coded according the three main categories (Armature = “AR”; Endscraper = “ES”; Borer = “BR”) and individual files are linked by naming conventions to their associated time slice, taxonomic unit, site, and literature source.

Note that for the purpose of the present analysis, these broader categories (AR, ES, BR) were not further disintegrated into individual lithic types, morphotypes or techno-types commonly distinguished in the literature.

Only complete or confidently reconstructed pieces have been included in the outline dataset for lithic armatures (AR) and endscrapers (ES), but not for borers (BR) because in the latter case the analytical aim of the data collection was not to analyse whole-outline variability but the shape configuration of borer working-ends (open outlines).

This is the general structure of the employed naming convention:

**Timeslice_TaxonomicUnit_Macroregion_KeySite_LiteratureSource_ToolCat_Axiality_SubID1(_SubID2)**

Here is a break-down of the different parts of this label structure and how they are expressed in the naming individual image files:

| Time-slice | “TS1”, “TS2”, “TS3” or “TS4” for Time-slice I, II, III or IV; image data that relates to multiple Time-slices is coded in the following way: Time-slices 1-2 = “TS12”, Time-slices 3-4 = “TS34” or Time-slices 2-4 = “TS234” |
| --- | --- |
| TaxonomicUnit | Identifier taken from “TaxUnit_unique” (see above) |
| Macroregion | Identifier taken from “Macro_region_code” (see above) |
| KeySite | Identifier taken from “Site_ID” (see above) |
| LiteratureSource | Short literature reference without spaces, e.g. “Mayer2000”; in case of multiple authors, e.g. “WebsterHut1998” or “Greenwoodetal2003” |
| ToolCat | Code for overarching/main tool categories mentioned above: “AR” for Armature, “ES” for Endscrapers, and “BS” for Borers (including Zinken) |
| Axiality | Axiality of points/orientation of tool axis in relation to blank axis: “d” = distal point (point axis aligns with blank axis), “p” = proximal point (point axis inversed in relation to blank axis), or “na” = not available/not determinable |
| SubID1 | Sub-id assigned to image files in numerical order that share all previous information |
| SubID2 | Sub-id assigned to individualized object-outlines in numerical after automated outline extraction |

**References**

Matzig, D.N. 2021a. outlineR: An R package to derive outline shapes from (multiple) artefacts on JPEG images. Zenodo. <https://doi.org/10.5281/ZENODO.4527469>.

Matzig, D.N. 2021b. outlineR: Artefact Processing and Extraction Protocol v1. DOI:dx.doi.org/10.17504/protocols.io.bygaptse (external link: [https://www.protocols.io/view/outliner-artefact-processing-and-extraction-protoc-rm7vz3pp8gx1/v1 09/22/2021](https://github.com/yesdavid/outlineR1%2009/22/2021)).
