# Supplementary material for: A quantitative analysis of Final Palaeolithic/earliest Mesolithic cultural taxonomy and evolution in Europe
Source: PLoS One. 2024 Mar 11;19(3):e0299512. doi: 10.1371/journal.pone.0299512 (PMC10927100; doi:10.1371/journal.pone.0299512)
Supplement: S5 Data — (DOCX) [file pone.0299512.s005.docx]

**ELECTRONIC SUPPLEMENTARY MATERIALS of**

*Riede et al., A quantitative analysis of Final Palaeolithic/earliest Mesolithic cultural taxonomy and evolution in Europe*

************************************************************

Supplementary Information S5: Additional/detailed results and analytical parameters

Additional parameters and calculated data as well as the constructed distance matrices used to assess within and between-group similarities in the analysed *Tools*, *Technology* and *Outlines* datasets are presented here.

1. ***Tools and Technology: Detailed CART results***

**S5 Table 1**. CART results for *Tools* (Partial data/Minimum error pruning).

| Level of Separation | Trait-State | N | Magdalenian | Epigravettian | ABP/ Azilian | ABP/ FMG | TPC/ LTP | FBT/ LBI | Mesolithic |
| --- | --- | --- | --- | --- | --- | --- | --- | --- | --- |
| FORK 1 | **Small tanged points (1)** | 21 | 14% | 0 | 0 | 14% | **57%** | 14% | 0 |
|  | **Small tanged points (0)** | 63 | 32% | 8% | 21% | 17% | 2% | 6% | 14% |
| FORK 2 | **Geometric triangles (1)** | 16 | 25% | 6% | 19% | 0 | 0 | 0 | **50%** |
|  | **Geometric triangles (0)** | 47 | 34% | 9% | 21% | 23% | 2% | 9% | 2% |
| FORK 3 | **Notched pieces (1)** | 20 | **65%** | 0 | 25% | 10% | 0 | 0 | 0 |
|  | **Notched pieces (0)** | 27 | 11% | 15% | 19% | 33% | 4% | 15% | 4% |
| FORK 4 | **Arched points (1)** | 19 | 5% | 21% | 26% | **47%** | 0 | 0 | 0 |
|  | **Arched points (0)** | 8 | 25% | 0 | 0 | 0 | 13% | **50%** | 13% |

**S5 Table 2**. CART results for Technology (Partial data/Minimum error pruning).

| Level of Separation | Trait-State | N | Magdalenian | Epigravettian | ABP/ Azilian | ABP/ FMG | TPC/ LTP | FBT/ LBI | Mesolithic |
| --- | --- | --- | --- | --- | --- | --- | --- | --- | --- |
| FORK 1 | ***En éperon* platform configuration (1)** | 12 | **100%** | 0 | 0 | 0 | 0 | 0 | 0 |
|  | ***En éperon* platform configuration (0)** | 72 | 15% | 7% | 18% | 19% | 18% | 10% | 13% |
| FORK 2 | **Tool production dependent on/follows blank production (1)** | 44 | 11% | 5% | 11% | **25%** | **30%** | 11% | 7% |
|  | **Tool production dependent on/follows blank production (0)** | 28 | **21%** | 11% | **29%** | 11% | 0 | 7% | **21%** |
| FORK 3a (TP_dep “1”) | **Retouch is occasional (1)** | 27 | 11% | 0 | 15% | **37%** | 19% | 7% | 11% |
|  | **Retouch is occasional (0)** | 17 | 12% | 12% | 6% | 6% | **47%** | 18% | 0 |
| FORK 3b (TP_dep “0”) | **Laminar production involves multidirectional reduction (1)** | 12 | 0 | 17% | 17% | 17% | 0 | 0 | **50%** |
|  | **Laminar production involves multidirectional reduction (0)** | 16 | **38%** | 6% | **38%** | 6% | 0 | 13% | 0 |

1. ***Outline similarity assessment***

To detect patterns in the 2D shape-data of the lithic artefacts, extracted artefact AR outlines were first described by Elliptic Fourier Analysis (EFA) and the retrieved harmonics then subjected to Principal Component Analysis (PCA). The obtained PC scores were then used as data to construct a Euclidian distance matrix to assess the similarity between individual object outlines. Included are also the p-values for significant statistical difference obtained for the stratified sub-samples of armature (AR) outlines compared across time-slices.

*p-Values of AR disparity analysis*

**S5 Table 3**. Mean sum of variances (disparity).

| Time-slice | n | mean | median | 2.5% | 25% | 75% | 97.5% |
| --- | --- | --- | --- | --- | --- | --- | --- |
| I | 867 | 0.044 | 0.043 | 0.039 | 0.042 | 0.045 | 0.050 |
| II | 1096 | 0.050 | 0.050 | 0.044 | 0.047 | 0.052 | 0.057 |
| III | 1123 | 0.071 | 0.071 | 0.062 | 0.068 | 0.074 | 0.081 |
| IV | 1155 | 0.112 | 0.112 | 0.101 | 0.108 | 0.116 | 0.123 |

**S5 Table 4**. Results of the pairwise Mann-Whitney test with Bonferroni correction.

| Comparison | W statistic | p-value |
| --- | --- | --- |
| I : II | 89899 | 0 |
| I : III | 0 | 0 |
| I : IV | 0 | 0 |
| II : III | 131 | 0 |
| II : IV | 0 | 0 |
| III : IV | 0 | 0 |
