# Supplementary material for: A quantitative analysis of Final Palaeolithic/earliest Mesolithic cultural taxonomy and evolution in Europe
Source: PLoS One. 2024 Mar 11;19(3):e0299512. doi: 10.1371/journal.pone.0299512 (PMC10927100; doi:10.1371/journal.pone.0299512)
Supplement: Peer review history

# Editor report: Decision Letter 0

**DOI:** 10.1371/journal.pone.0299512.r001

**Authors:** Marco Peresani

12 Feb 2024

A quantitative analysis of Final Palaeolithic/earliest Mesolithic cultural taxonomy and evolution in Europe

PONE-D-23-41635

Dear Dr. Riede,

We’re pleased to inform you that your manuscript has been judged scientifically suitable for publication and will be formally accepted for publication once it meets all outstanding technical requirements.

This work is an outstanding excellent contribution for shedding light to the variability of the archaeological record at a macro-regional level in Western and Central Europe. Thanks to the accessibility of data, the authors make possible to replicate their analysis, despite the deprecability of packages. It will be of reference for scholars investigating on the reality of discrete archaeological cultural units and their implication in the reconstruction of the bio-cultural evolution of last Pleistocene – Early Holocene hunter-gatherers. The manuscript, figures, tables and outlined data have been thoroughly revised by the authors after a first round of reviews in PCI https://doi.org/10.24072/pci.archaeo.100388 and recommended by Anais Vignoles. After my examination of the revised tracked manuscript, authors’ response to reviewers and the amended manuscript, I am convinced that this contribution will be considered in discussions focusing on the reliability of some European Late Paleo/Early Mesolithic cultural entities, as well as on the epistemological and historical factors affecting their taxonomy. Therefore, this manuscript received by Riede and colleagues’ manuscript is worthy to be accepted for publication. 

Within one week, you’ll receive an e-mail detailing the required amendments. When these have been addressed, you’ll receive a formal acceptance letter and your manuscript will be scheduled for publication.

An invoice for payment will follow shortly after the formal acceptance. To ensure an efficient process, please log into Editorial Manager at http://www.editorialmanager.com/pone/, click the 'Update My Information' link at the top of the page, and double check that your user information is up-to-date. If you have any billing related questions, please contact our Author Billing department directly at authorbilling@plos.org.

If your institution or institutions have a press office, please notify them about your upcoming paper to help maximize its impact. If they’ll be preparing press materials, please inform our press team as soon as possible -- no later than 48 hours after receiving the formal acceptance. Your manuscript will remain under strict press embargo until 2 pm Eastern Time on the date of publication. For more information, please contact onepress@plos.org.

Kind regards,

Marco Peresani

Academic Editor

PLOS ONE

Journal requirements:

When submitting your revision, we need you to address these additional requirements.

1. Please include captions for your Supporting Information files at the end of your manuscript, and update any in-text citations to match accordingly. Please see our Supporting Information guidelines for more information: http://journals.plos.org/plosone/s/supporting-information.

# Editor report: Acceptance letter

**DOI:** 10.1371/journal.pone.0299512.r002

**Authors:** Marco Peresani

26 Feb 2024

PONE-D-23-41635 

PLOS ONE

Dear Dr. Riede, 

I'm pleased to inform you that your manuscript has been deemed suitable for publication in PLOS ONE. Congratulations! Your manuscript is now being handed over to our production team.

At this stage, our production department will prepare your paper for publication. This includes ensuring the following:

* All references, tables, and figures are properly cited

* All relevant supporting information is included in the manuscript submission,

* There are no issues that prevent the paper from being properly typeset

If revisions are needed, the production department will contact you directly to resolve them. If no revisions are needed, you will receive an email when the publication date has been set. At this time, we do not offer pre-publication proofs to authors during production of the accepted work. Please keep in mind that we are working through a large volume of accepted articles, so please give us a few weeks to review your paper and let you know the next and final steps. 

Lastly, if your institution or institutions have a press office, please let them know about your upcoming paper now to help maximize its impact. If they'll be preparing press materials, please inform our press team within the next 48 hours. Your manuscript will remain under strict press embargo until 2 pm Eastern Time on the date of publication. For more information, please contact onepress@plos.org.

If we can help with anything else, please email us at customercare@plos.org.

Thank you for submitting your work to PLOS ONE and supporting open access. 

Kind regards, 

PLOS ONE Editorial Office Staff

on behalf of

Dr. Marco Peresani 

Academic Editor

PLOS ONE
